# Microglia induce neurogenic protein expression in primary cortical cells by stimulating PI3K/AKT intracellular signaling in vitro

**DOI:** 10.1007/s11033-020-06092-0

**Published:** 2021-01-02

**Authors:** Kristi Lorenzen, Nicholas W. Mathy, Erin R. Whiteford, Alex Eischeid, Jing Chen, Matthew Behrens, Xian-Ming Chen, Annemarie Shibata

**Affiliations:** 1 Biology Department, Creighton University, Omaha, NE USA; 2 Department of Medical Microbiology and Immunology, Creighton University School of Medicine, Creighton University, Omaha, NE USA; 3 University of Nebraska Medical Center, Omaha, NE USA; 4 Pediatric Medicine, St. Joseph Heritage Healthcare, Chino Hills, CA USA; 5 Stanford Hospital and Clinics, 300 Pasteur Dr, Stanford, CA USA; 6 University of Nebraska College of Medicine, Omaha, NE USA

**Keywords:** Microglia, Nestin, α-internexin, Neurogenesis, PI3K/AKT

## Abstract

Emerging evidence suggests that microglia can support neurogenesis. Little is known about the mechanisms by which microglia regulate the cortical environment and stimulate cortical neurogenesis. We used an in vitro co-culture model system to investigate the hypothesis that microglia respond to soluble signals from cortical cells, particularly following mechanical injury, to alter the cortical environment and promote cortical cell proliferation, differentiation, and survival. Analyses of cortical cell proliferation, cell death, neurogenic protein expression, and intracellular signaling were performed on uninjured and injured cortical cells in co-culture with microglial cell lines. Microglia soluble cues enhanced cortical cell viability and proliferation cortical cells. Co-culture of injured cortical cells with microglia significantly reduced cell death of cortical cells. Microglial co-culture significantly increased Nestin + and α-internexin + cortical cells. Multiplex ELISA and RT-PCR showed decreased pro-inflammatory cytokine production by microglia co-cultured with injured cortical cells. Inhibition of AKT phosphorylation in cortical cells blocked microglial-enhanced cortical cell viability and expression of neurogenic markers in vitro. This in vitro model system allows for assessment of the effect of microglial-derived soluble signals on cortical cell viability, proliferation, and stages of differentiation during homeostasis or following mechanical injury. These data suggest that microglia cells can downregulate inflammatory cytokine production following activation by mechanical injury to enhance proliferation of new cells capable of neurogenesis via activation of AKT intracellular signaling. Increasing our understanding of the mechanisms that drive microglial-enhanced cortical neurogenesis during homeostasis and following injury in vitro will provide useful information for future primary cell and in vivo studies.

**Supplementary Information:**

The online version contains supplementary material available at 10.1007/s11033-020-06092-0.

## Introduction

Microglia are resident immunocompetent and phagocytic cells of the central nervous system (CNS) and comprise anywhere from 5–12% of cortical cells [45, 63]. During homeostasis, microglia survey the local CNS environment and communicate with neighboring glia and neurons through membrane-bound and soluble signals [59, 68]. Emerging evidence suggests that, given specific activator(s), microglia function to support proliferation, differentiation, synaptic function, and survival of neurons [68, 70].

Interestingly, microglial invasion of the cortical plate overlaps with peak periods of cortical neurogenesis [59, 63, 78]. After invasion, microglia remain as morphologically and functionally dynamic cells within the environment of the cortex [28, 45, 59]. Microglial-derived cytokines may promote neurogenesis by supporting progenitor cell survival and mitosis [51, 57, 72]. The extent to which microglia can promote neurogenesis may depend upon their state of activation [10, 38]. Microglia contribute to synaptic development by refining axonal branching and pruning synaptic connections through phagocytic activity [41, 47, 66, 85]. Additionally, specific microglial-derived cytokines, growth factors, and cell associated proteins may play an important role in the modification and function of both excitatory and inhibitory synaptic connections in the CNS [11, 54, 62, 71]. Microglia support neurogenesis in the classic neural stem cell niches of the subgranular zone of the dentate gyrus in the hippocampus and the subventricular zone lining the lateral ventricles [3, 16, 17, 20, 48, 72, 82]. Microglial proliferation and increased release of transforming growth factor –β (TGF-β) are correlated with neural stem cell proliferation in the adult dentate gyrus [54]. Secretion of insulin growth factor 1 (IGF-1) from microglia following status epilepticus in the adult dentate gyrus stimulates neurogenesis via activation of the p42/44 MAPK pathway [84]. Other studies suggest that injury to adult CA1 neurons of the dentate gyrus stimulates IGF-1 release from microglia and astrocytes promoting neuronal survival via AKT phosphorylation and decreased MAPK phosphorylation [80] or via both AKT and MAPK phosphorylation [73].

While an ever-growing body of work supports the role of microglial soluble signals in proper neurogenesis and plasticity [15, 75], microglial proinflammatory soluble signals are also linked to neurotoxicity and neurodevelopmental and neurodegenerative diseases [19, 69]. As such, a reduction in microglial-derived neuroinflammatory cytokines such as TNF-α, IFN-γ, MIP-1α and RANTES/CCL5, IL-1α, and IL-1β has been shown to suppress apoptosis and enhance neurogenesis [6, 9, 10]. Production of anti-inflammatory mediators and neurotrophic factors by microglia are likely to be dependent on the nature and duration of the stimulus as well as the severity of injury to which microglia respond. Recent work demonstrates that primary microglia, even in the same region of the brain, express complex patterns of gene expression resulting in functionally diverse microglial phenotypes [26, 28]. Taken together, these data suggest that a complex milieu of microglial-derived soluble cues with neurogenic or neuroinflammatory properties work in combination to promote or restrict neuronal development, survival, and repair following injury.

Given the diverse functional roles that microglia can play in the cortex, the development of an in vitro model system to evaluate the neurogenic potential of a uniform population of microglia is important. We hypothesize that microglia can stimulate localized cortical neurogenesis during homeostasis or following injury in the cortex by activating specific intracellular signaling pathways required for neuronal survival and differentiation. Little is known about the mechanisms by which homeostatic microglia or activated microglia responding to mechanical injury in the cortex influence neurogenesis and survival. We have utilized an in vitro co-culture system comprised of microglial cell lines suspended above primary cortical cells that were mechanically injured or left uninjured as control. Neurogenesis in primary cortical cell culture can be determined by evaluating the progressive expression of neurogenic proteins such as Nestin, GFAP, α-internexin, and NeuN [27, 46, 88]. Nestin expression has long been known to be upregulated in neural progenitor cells and cortical radial glia [40, 53]. Several studies also report that Nestin protein is expressed in early differentiating neurons in rodents and humans [30, 43, 81]. Nestin and doublecortin immunopositive cells have been shown to be responsive to injury in the spinal cord [18]. Recently, Nestin has been shown to be co-expressed with doublecortin in immature neurons of the cortex [7]. The regulation of Nestin expression in the cortex in response to injury and immune signaling is not well known. GFAP expression is associated with early stages of neurogenesis and is co-expressed with Nestin in neural stem cells and in the cells of cortical parenchyma following injury [61]. GFAP is also expressed in populations of mature astrocytes [74] although astrocyte populations are quite diverse [49]. Later in neurogenesis, cytoskeletal elements such as α-internexin and neurofilament are upregulated and highly expressed as neurons begin to mature [5, 36]. Alpha-internexin is expressed in postmitotic immature neurons as they [36]. The neuronal splicing regulator, NeuN, is expressed in both post-mitotic immature and mature cortical neurons [27]. These well characterized stages of neurogenesis in primary cortical cells were used to assess the effect of microglia soluble cues on cortical cells during homeostasis and following cortical cell mechanical injury.

Our data suggest that microglial cell line-derived soluble cues promote cortical cell viability and enhance proliferation of cortical cells. Microglial-derived cues reduced cell death of primary cortical cells following acute mechanical injury in vitro. In injured cortical cell and microglial co-cultures, significantly increased expression of neurogenic markers Nestin and α-internexin was present within the site of injury where proliferating cells were observed. Expression of the mature neuronal marker NeuN increased in injured cortical cells outside the injury site when co-cultured with microglial cells. AKT phosphorylation was increased in cortical cells co-cultured with EOC2 microglial cells. Inhibition of AKT phosphorylation reduced the enhanced expression of neurogenic markers in cortical cell and microglial co-cultures. EOC2 microglial cells responding to acute injury cortical cell injury downregulated their expression of pro-inflammatory cytokine mRNA and protein. This co-culture system provides a useful tool to further investigate the neuroimmune mechanisms important for primary microglial responses and cortical cell differentiation and survival in vitro following injury and informs future in vivo experimentation.

## Materials and methods

### Isolation of primary cortical cells

Female timed-pregnant Sprague Dawley rats (200–250 g) were purchased from Charles River Laboratories (USA). Use of animals was performed in strict accordance with Institutional Animal Care and Use committee guidelines as approved by the IACUC committee at Creighton University (protocol #0793). Timed Sprague Dawley dams were housed for up to 3 days in Creighton’s Animal Resource Facility that is AALAC accredited. Ad libitum food and water and normal 24 h light/dark schedules were followed. Cortical cultures were established as described previously [52]. Briefly, dams were euthanized by CO_2_ asphyxiation. E16-E18 embryos were removed from their placental sacs and immediately decapitated. Brains were removed, meninges were removed, and cerebral cortices were dissected. Cortical tissue was mechanically dissociated in Ca^2+^/Mg^2+^-free Hank’s balanced salt solution, with 0.035% sodium bicarbonate and 1 mM pyruvate (pH 7.4) and digested for 15 min with 2.5% trypsin at 37 °C. Trypsin was neutralized with Dulbecco’s Modified Eagles Media (DMEM: Hyclone, Thermofisher Scientific, Waltham, MA) plus 10% fetal bovine serum. The cell suspension was washed three times, triturated, and resuspended with neurobasal media supplemented with B-27® and penicillin/streptomycin (Thermofisher Scientific, Waltham, MA). Cells were plated onto poly-D-lysine coated plates and coverslips (Sigma, St. Louis, MO) at a density of 1.5 × 10^6^ cells/well in 6-well plates and 5 × 10^5^ cells/well in 24-well plates and were maintained at 37 ºC in 5% CO_2_ in neurobasal supplemented media. Each cortical culture from 1 pregnant dam was considered to be a biological replicate. Embryonic brain tissue was used for co-culture, immunocytochemistry, multiplex ELISA, RT-PCR and western blot experiments all performed in triplicate. In total, 24 Sprague Dawley dams were used for these data.

### Cultivation of microglia

EOC 2 microglia isolated were purchased from American Type Culture Collection (ATCC CRL-2467; Manassas, VA) and were maintained in DMEM (Hyclone, Thermofisher Scientific, Waltham, MA) plus 10% fetal bovine serum, 1% l-glutamine, 1% penicillin/streptomycin, and 20% LADMAC conditioned media. Cells were grown in 100-mm tissue culture dishes at 37 ºC in 5% CO_2_ and allowed to reach 80% confluency before the cells were passed. LADMAC conditioned media (as a source of CSF-1) was collected from *Mus musculus* bone marrow derived LADMAC cells (ATCC CRL-2420; Manassas, VA) 5–7 days after initial plating of cells at 1 × 10^5^ in Eagle’s Minimal Essential Media (MEM; Hyclone) supplemented with 10% Fetal Bovine Serum (FBS, Alas Biologicals, Ft. Collins, CO), 1% l-glutamine, 0.1 mM nonessential amino acids, 1.0 mM sodium pyruvate, and 1% penicillin/streptomycin. BV2 microglial cells were a generous gift from Dr. Xian Ming Chen. BV2 microglia were sub-cultured in DMEM (Hyclone, Thermofisher Scientific, Waltham, MA) supplemented with 10% fetal bovine serum, 1% l-glutamine, 1% penicillin/streptomycin and maintained as described for EOC2 cells.

### Neuronal – microglial co-cultures

Primary cortical cells were cultured for 48 h then either injured by mechanical transection using a sterile stylet [55] or left uninjured. Briefly, mechanical transection using a sterile stylet involved the application of the sterile stylet tip directly to the cortical cell culture. Pressure was placed on the stylet while dragging the stylet tip across the cortical cell culture to form parallel sites of injury in the cortical culture. Microglia were pre-seeded directly onto 6-well permeable 0.40 µm Transwells® at 5 × 10^5^ cells/well or onto 24-well Transwells® at 4 × 10^4^ cells/well (Corning, Tewksbury, MA) and cultured for 24 h before being suspended above cortical cells using Transwells® in the co-culture model system. Microglia seeded onto Transwells® and injured or uninjured (control) primary cortical cells were co-cultured for an additional 48 h in unsupplemented neurobasal media prior to cellular assays. These co-cultures are similar to those previously described by [79] but differ in the pore size of the Transwells®, mechanism of injury, and cell types used in the culture system.

### Immunocytochemistry

Primary cortical cultures were plated onto sterile poly-D-lysine coated coverslips. Microglia were plated onto sterile glass coverslips suspended above cortical cultures in Transwells®. Following co-culture experiments, cortical cells and microglia were fixed with 4% paraformaldehyde for 15 min at room temperature and washed with 1X PBS. Cells were permeabilized with 0.2% Triton X-100 in PBS for 10 min, washed, and blocked for 1 h in PBS, 0.2% BSA, and 0.2% Triton X-100. Primary antibodies were applied and incubated overnight at 4 ºC in PBS, 0.2% BSA, and 0.2% Triton X-100. Primary antibodies were purchased from RMD Millipore Sigma (Darmstadt, Germany) and included: mouse anti-Nestin (1:200, Millipore Cat# AB5922, RRID:AB_91107), rabbit anti-GFAP (1:400, Millipore Cat# AB5541, RRID:AB_177521), mouse anti-α-internexin (1:100, Millipore Cat# AB5354, RRID:AB_91800), mouse anti-TUJI/beta tubulin III (1:200, Millipore Cat# MAB1637, RRID:AB_2210524) and mouse anti-NeuN (1:50, Millipore cat#MAB377l, RRID:AB_2298772). The primary antibody directed against neurofilament was a mouse monoclonal neurofilament antibody, p-NF-H (7H11) (1:200, Santa Cruz Biotechnologies, Cat# sc-20015, RRID: AB_670161)**.** To confirm microglial characteristics, microglial cells were immunostained with rabbit anti-mouse CD11b conjugated to Alexa 488 (2 µg/100 µl, Caltag Laboratories, Burlingame, CA). Secondary antibodies were applied for 1 h at a concentration of 1:500 for goat anti-rabbit IgG (H + L) rhodamine conjugate and goat anti-mouse IgG (H + L) fluorescein conjugate (Pierce, Rockford, IL). Nuclei were visualized using a DAPI stain (300 mmol, MP Biomedicals, Santa Ana, CA). Qualitative and quantitative analysis of immunocytochemistry was performed by acquiring images with a Leica DMI4000B inverted microscope with a cooled CCD camera (Q Imaging, Surrey, BC) and fluorescent capabilities. Quantification of the percent of cells expressing neurogenic markers was determined by counting the number of immunopositive cells for each marker and dividing by the total number of cells counted in the field. Experiments were performed in triplicate with at least 300 cells counted/ experiment/condition. Quantification of relative fluorescence intensity units (RFU) was calculated by subtracting pixel intensity from the background immunofluorescence of each fluorochrome. Experiments were performed in triplicate with at least 300 cells counted/experiment/condition. In all experiments, images were analyzed with Volocity (PerkinElmer, USA), and ImageQuant (GE Healthcare, USA) and ImageQuant (GE Healthcare, USA) software were used for image analysis and presentation.

### Measurement of cell viability

Viability of cortical cells and microglia were measured by metabolism of thiazolyl blue, 3-[4,5-dimethylthiazol-2yl]-2,5-diphenyltetrazolium bromide (MTT, Sigma Aldrich) and CellTitre-Glo®(Promega, Thermo Fisher Scientific, Waltham, MA). For MTT, injured and uninjured cortical cells cultured with and without microglia were incubated with 100 µl MTT in 1 ml of media for 1 h. Media was removed and cells were dissolved in 300 µl dimethylsulfoxide (DMSO) and aliquoted to 100 µl/well in 96-well plates. Absorbance was read at 540-590 nm on an ELISA plate reader. Three experiments were performed in triplicate. For CellTitre-Glo® manufacturer’s protocol was followed (https://www.promega.com/-/media/files/resources/protocols/technical-bulletins/0/celltiter-glo-luminescent-cell-viability-assay-protocol.pdf?la=en). Briefly, injured and uninjured cortical cells cultured with and without microglia were incubated with pre-mixed CellTitre-Glo®, Reagent equal to the cell culture medium volume, cells and Reagent were misted for 2 min to allow for cellular lysis, and the plate was incubated for 10 min. For each condition triplicate 100 µl samples were placed into 96 well plates and chemiluminescence was recorded to measure ATP and metabolic activity of viable cells (BioTek Synergy, Winooski, VT, USA).

### Measurement of cortical cell proliferation

Cell proliferation was measured using Click-iT® EdU Alexa Fluor 647 according to the manufacturer’s instructions (C10340, Thermo Fisher Scientific, Waltham, MA). Briefly, Click-iT® EdU Alexa Fluor 647 is a modified thymidine analogue EdU (5-ethynyl-2′-deoxyuridine, a nucleoside analog of thymidine) that is incorporated into newly synthesized DNA. The EdU is fluorescently labeled with a photostable Alexa Fluor® dye during the click reaction. Uninjured and injured cortical cells co-cultured with and without microglia for 2 DIV (days in vitro) were fixed in 3.7% formaldehyde in PBS for 15 min at RT. Fixed cells were washed twice with 1 ml of 3% BSA in PBS. Cells were permeabilized in 0.5% Triton®x-100 for 20 min at RT, washed and 1X Click-iT® EdU reaction cocktail was added for 30 min at RT. The reaction cocktail was removed, cells were washed in 3% BSA and PBS, counterstained with DAPI, mounted and imaged for analysis. Imaging was performed using IBIF Leica TCS SP8 MP Confocal Microscope at 20 × magnification. Experiments were performed in triplicate with at least 300 cells counted manually per experiment for each condition. Volocity (PerkinElmer, USA) and ImageQuant (GE Healthcare, USA) software were used for image analysis and presentation.

### Measurement of cortical cell death

Cell death of cortical cultures was measured following co-culture with microglia for 2 DIV using a Click-iT® TUNEL Alexa Fluor 488 imaging assay (C10245, Thermo Fisher Scientific, Waltham, MA). Manufacturer’s instructions were followed. Briefly, injured and uninjured cortical cells cultured with or without microglia were fixed with 4% PFA in PBS for 15 min then permeabilized with 0.25% Triton-X® 100 for 20 min. Each condition was incubated with 100 µl of TdT reaction buffer for 10 min at RT then removed. Cells were incubated in 100 µl TdT reaction cocktail for 1 h  at 37 °C. Cells were washed twice in 3% BSA in PBS for 2 min then incubated with Click-iT® reaction buffer with additive for 30 min at RT protected from light. Cells were rinsed and counterstained with DAPI, mounted, and cover slipped for analysis. Imaging was performed using IBIF Leica TCS SP8 MP Confocal Microscope at 20 × magnification. Experiments were performed in triplicate with at least 300 cells counted manually per experiment for each condition. Volocity (PerkinElmer, USA) and ImageQuant (GE Healthcare, USA) software were used for image analysis and presentation.

### ELISA analysis

Conditioned media was collected from uninjured and injured neuronal and microglia co-cultures from three separate experiments and cytokine expression was determined by Q-Plex™ mouse cytokine –Inflammation multiplex assay. Concentrations of mouse microglia-derived cytokines MCP-1, IFN-γ, MIP-1α, TNFα, RANTES, IL-1α, IL-1β, IL-2, IL-4, IL-3, IL-6, IL-10, IL-12, 1L-17, and GM-CSF were evaluated by Quansys Biosciences (#110449MS, Logan, UT). Cytokine concentrations in media collected from uninjured cortical cells cultured with microglia were used as the reference and control for these experiments. In order to use cytokine concentrations from uninjured cortical and microglial co-cultures as our control condition, each cytokine concentration measured in the uninjured cortical cell and microglia co-culture condition was normalized and set equal to one. Cytokine concentrations in media collected from injured cortical cells co-cultured with microglia were measured, normalized, and expressed as the percent change in cytokine concentration as compared to uninjured control concentrations for that cytokine. Multiplex ELISA assays were run in triplicate in three biological replicate experiments. Significance of the percent change from control was determined using student T test with Bonferroni correction. The percent change in cytokine concentration was considered significant if p < 0.05 and error bars represent the standard error of the mean of the percent change.

### RT-PCR analysis

For real-time PCR analysis of cytokines, total RNA of EOC2 microglial cultured on Transwell® permeable inserts that were physically separated from cortical cells was extracted using the mirVana miRNA Isolation kit (Ambion). An amount of 200 ng total RNA was reverse-transcribed using the Invitrogen™ NCode™ miRNA First-Strand cDNA Synthesis Kit (Thermo Fisher Scientific). Comparative real-time PCR was performed using the Invitrogen™ SYBR GreenER™ qPCR SuperMix Universal (Thermo Fisher Scientific) on the Bio-Rad CFX96 Touch™ Real-Time PCR Detection System. Primers were purchased from QIAGEN (Ccl3, Ccl5, Ifnγ, Mcpt1, Tnfα, Gapdh). Normalization was performed using GAPDH. Relative expression was calculated using the comparative Ct (ΔΔCt) method.

#### Western blots

Following co-culture, protein collected from cortical cells was assessed using western blot analysis. Cortical cells were lysed with 500 µl lysis buffer (10 × lysis buffer, Cat#9803, Cell Signaling, Danvers, MA), supplemented with 0.1 M PMSF (Cat # 36,978, Thermo Fisher Scientific, Waltham, MA), and HALT™ protease and phosphatase inhibitor diluted to 1X (Cat#78,446, Thermo Fisher Scientific, Waltham, MA) per 3 wells of the 6-well plates. Lysates were spun 10,000 RPM for 10 min at 4 °C. Lysate supernatant were heated at 95 °C for 5 min with 4X sample buffer plus 10 mM DTT. Denatured protein samples were separated by SDS-PAGE gel electrophoresis on 10% TGS gels. Proteins were transferred to PVDF membranes in Tris–glycine transfer buffer. After transfer, membranes were blocked using BSA Blocking Buffer™ in TBS (Cat#37,520, Thermo Fisher Scientific, Waltham, MA) for 1 h and then incubated with primary antibody diluted in BSA Blocking Buffer™ in TBS overnight at 4 °C. Primary antibodies included rabbit anti-phospho-p44/42 MAPK (1:1000, 1:2000, Cell Signaling Technology Cat# 4376, RRID:AB_331772), rabbit anti-pan AKT (1:1000, Cell Signaling Technology Cat# 4691, RRID:AB_915783) rabbit anti-phospho-AKT (1:100, Cell Signaling Technology Cat# 9270, RRID:AB_329824). Following washing in Tris Buffered Saline with 0.1% Tween® 20 and BSA blocking buffer™, appropriate secondary antibodies (anti-rat IgG, HRP-linked antibody,1:1000, Cell Signaling Technology, Danvers, MA) were applied for detection. Membranes were developed using chemiluminescence SuperSignal™ ELISA Pico Chemiluminescent Substrate (Cat#37,069, Thermo Fisher Scientific, Waltham, MA) following manufacturer’s instructions. Statistical analyses involved semi-quantitative measurements of chemiluminescence using BioRad ChemiDoc QRS (Hercules, CA) imaging system and software. Total protein loading was assessed by detecting GAPDH in each sample. Three separate experiments were performed for measurements of protein expression by densitometry.

#### Signal transduction inhibitor assays

Primary cortical and microglial co-cultures were established as described above. Stock solutions of kinase inhibitors in DMSO were prepared at stock concentrations recommended by the manufacturer. Stocks were stored at − 20 °C and diluted into cell culture media prior to use. Four hours prior to injury and co-culture with microglia, signaling pathway inhibitors were added at concentrations of 0 µM, 10 µM, or 40 µM to the cortical cultures. The following inhibitors were tested: MAPK inhibitor PD98059 (Cat#9900S), PI3K/AKT inhibitor LY294002 (Cat#9901S), PKC and Glycogen synthase kinase-3 inhibitor GF109203X (Cat#984,150), Janus kinase 2 inhibitor AG490 (Cat#14704S). All inhibitors were purchased from Cell Signaling Technologies (Danvers, MA). Uninjured and injured cortical cells that were not cultured with microglia were used as controls. For control experiments, DMSO vehicle diluted in culture media was used in the experiments. After 48 h, cells were analyzed using MTT assays (see above) or fixed with 4% PFA in PBS to observe expression for neurogenic markers, Nestin, α-internexin, and GFAP using immunocytochemical methods as described above. To quantify imaging data, 3 field views of at least 100 cells from 3 separate experiments were analyzed for each condition.

#### Immunoprecipitation for AKT/pAKT analysis

Immunoprecipitation for AKT and pAKT was used to increase specificity and detection of AKT protein in cellular lysates. Cellular cultures were lysed as described above and each condition was split into two aliquots (200 µl each). Primary antibodies AKT (pan) (C67E7) rabbit mAB (Cell Signaling Technology Cat# 4691, RRID:AB_915783) and Phospho- AKT (Thr308) rabbit mAB (Cell Signaling Technology Cat#9275, RRID:AB_329828) were added at 1:50 for each sample and rotated overnight at 4 °C. A 50% slurry of EZview Red Protein A Affinity Gel Beads (Cat#P6486, EDM Millipore Sigma, Darmstadt, Germany) were added at 1:10 for each sample and rotated for 1 h at 4 °C. Cells were centrifuged at 8200* g* for 1 min and washed with lysis buffer 3 times. Samples were heated at 95 °C for 5 min with 25 µl 3X sample buffer. Samples were run on 4–20% gradient SDS–polyacrylamide gels (Cat#4,561,096, BioRad, Hercules, CA) using SDS-PAGE and then transferred to PVDF membrane. Membranes were blocked using BSA Blocking Buffer™ in TBS for 1 h and then gently rocked with the primary AKT or pAKT antibody at 1:1000 in BSA Blocking Buffer™ in TBS overnight at 4 °C. Blots were washed and incubated in secondary anti-rabbit HRP conjugated antibody for 1 h at RT. Membranes were developed using chemiluminescence as described above. Three separate experiments were performed. Statistical analysis involved analysis of densitometric images acquired with BioRad ChemiDoc QRS imaging system and software (BioRad, Hercules CA) which were performed as described above.

#### Statistical analysis

Data are expressed as mean values and error bars represent standard error of the mean (SEM). Student T test with Bonferroni’s correction or one-way ANOVA followed by Tukey–Kramer post hoc tests were performed where appropriate. For determination of significant differences between percents and for multiple comparisons between culture conditions, one-way or two-way ANOVA followed by Tukey–Kramer multiple analyses post hoc tests were used. Values of p < 0.05 were considered to be significant. All statistical analyses were performed with Graphpad Prism 8 (La Jolla, CA).

## Results

**Fig. 1:**
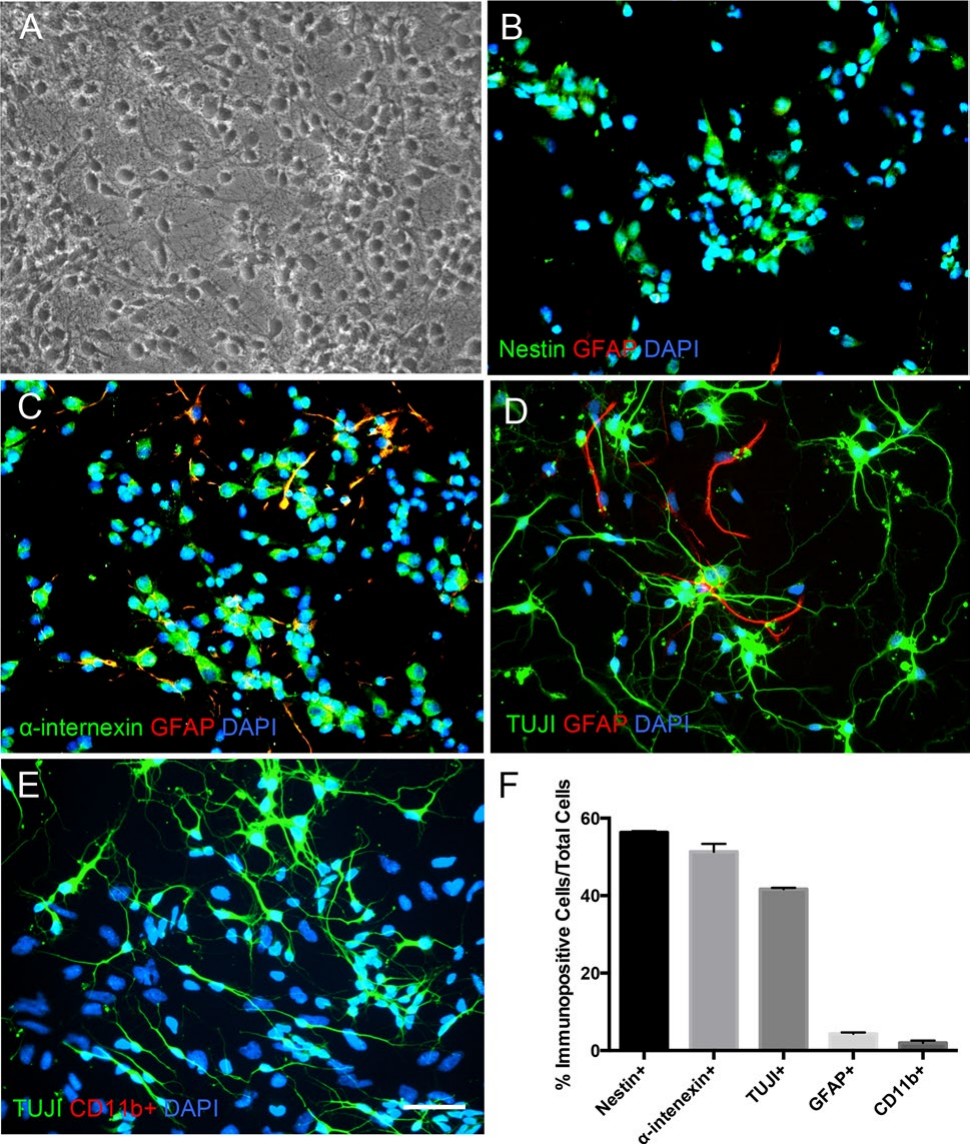
Primary cortical cells in the in vitro system. **a** Phase contrast image of cortical cells in primary culture. Cells have rounded cell bodies and extension of processes is visible. **b** Fluorescent image of primary cortical cells of Nestin + and GFAP + cells. **c** Fluorescent image of primary cortical cells showing α-internexin + and GFAP + cells. **d** Fluorescent image of primary cortical cells showing TUJI + and GFAP + cells. **e** Fluorescent image of primary cortical cultures showing CD11b + and GFAP + cells. DAPI (Blue) was to identify nuclei of all cells within an imaged field. Scale bar represents 50 µm. All images were taken with a 20X Leica objective. **f** Quantification of immunopositive cells. Percent of cells immunopositive for Nestin, α-internexin + , GFAP, and TUJI was calculated for three separate fields with each field having at least 100 cells. Error bars represent SEM. (Color figure online)

In vitro co-cultures were established to investigate the effect of microglial soluble cues on cortical cell proliferation, survival, and differentiation during homeostasis and following cortical cell mechanical injury. To characterize the primary cortical cell types, immunocytochemical analysis was performed at two days in vitro (2 DIV, Fig. 1). In control cortical cultures, 56.3 ± 0.3% were Nestin + , 51.3 ± 2.0% were α-internexin + , 41.7 ± 0.3% were TUJI + , and 4.3 ± 0.3% were glial fibrillary acidic protein (GFAP) + (Fig. 1b-e). Only 1.9 ± 0.6% were immunopositive for the microglial marker CD11b (CD11b +) demonstrating that the culture conditions did not support primary microglial cell proliferation and survival (Fig. 1e).

**Fig. 2:**
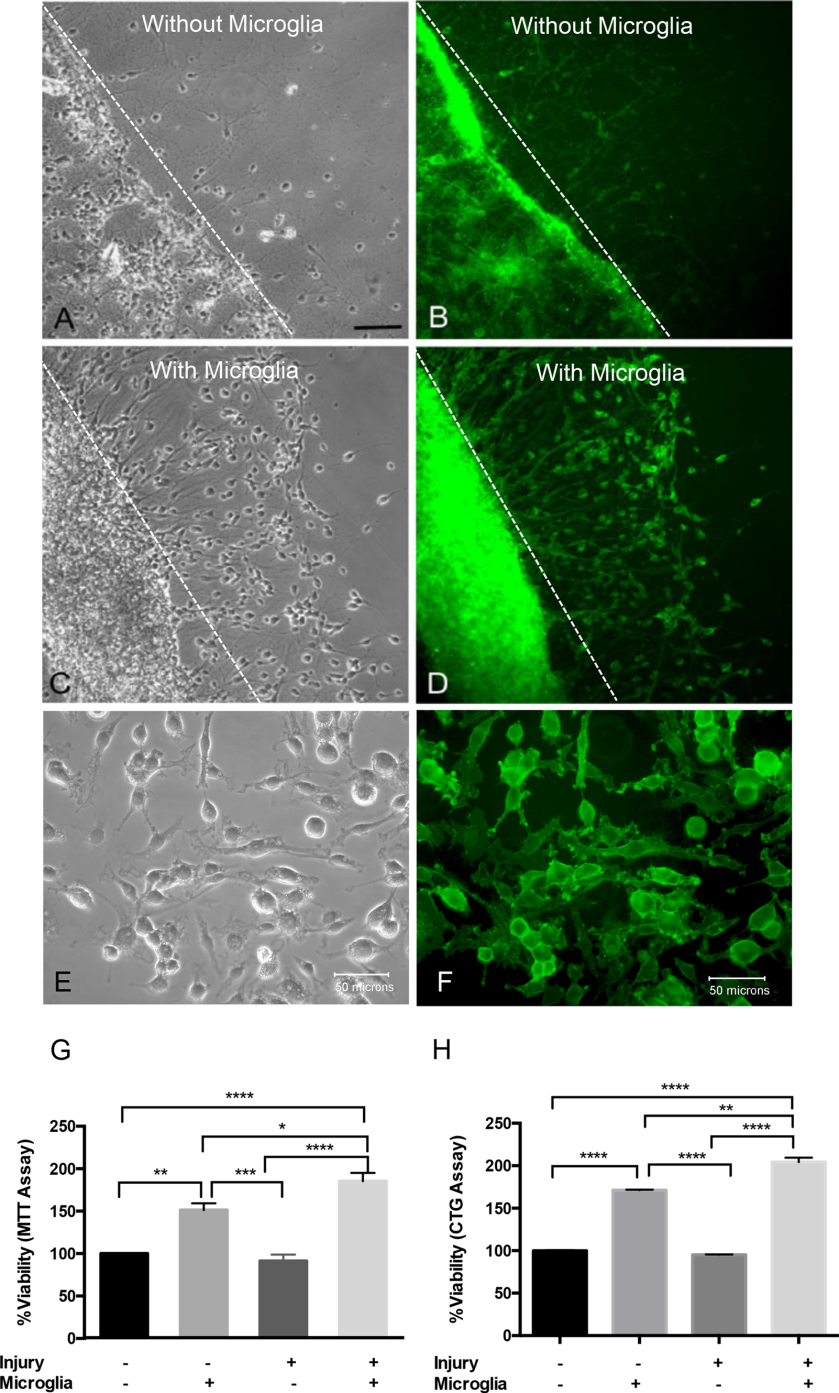
Phase contrast and fluorescent images of EOC2 microglial co-culture with injured primary cortical cells. **a**. Phase contrast image of primary cortical cells following mechanical injury mediated by stylet transection. No microglia were suspended above the injured neurons. The dashed white line indicates the site of injury. Black scale bar represents 100 µm. **b** Fluorescent image of neurofilament (NF +) cortical cells 2 DIV following injury. NF + immunoreactivity indicates the location of cortical cells in primary cortical cultures 2 DIV following injury. Immunoreactivity of NF + cells was seen primarily at the site of injury. Microglia were not suspended above this cortical cell culture. **c** Phase contrast image of primary cortical cells following injury and co-culture with microglia for 2 DIV. A dramatic increase in the number of cortical cells was visible at and within the site of injury (dashed white line represents the site of injury). **d** Fluorescent image of NF + cortical cells in microglia co-culture 2 DIV following injury. Dashed white indicates the site of injury. Immunoreactivity for NF + was seen within the site of injury when cortical cells were co-cultured with microglia. **a**–**d** Images were taken with the 20X Leica objective. **e** Phase contrast images of cultured EOC2 microglia cells co-cultured with primary neurons on 0.40 µm Transwell® inserts. These inserts were suspended directly above cortical cells. **f** Microglia used in this co-culture system were immunopositive for the microglial marker CD11b-Alexa 488 (green immunofluorescence) antigen. **e**–**f** Scale bar represents 50 µm. Images were taken with the 40X Leica objective. **g** Viability of uninjured cortical cells or injured cortical cells with or without microglial co-culture for 2 DIV was assessed using the MTT assay. MTT activity was read at OD 595 nm for three separate experiments. OD data was converted to percent viability and compared to uninjured control cortical cell viability that served as control. Error bars representing SEM. One-way ANOVA followed by Tukey’s multiple comparisons test was performed to determine the significance and yielded a F value = 44.0. Significance is *p < 0.05, **p  0.01, ***p < 0.001, ****p < 0.0001). **h** Viability of uninjured cortical cells or injured cortical cells with or without microglial co-culture for 2DIV was assessed using the CellTitre-Glo (CTG) assay. CTG chemiluminescent activity was read for three separate experiments. Chemiluminescence was converted to percent viability and compared to uninjured control cortical cell viability that served as control. Error bars representing SEM. One-way ANOVA followed by Tukey’s multiple comparisons test was performed to determine the significance and yielded a F value = 462.8. Significance is *p < 0.05, **p < 0.01, ***p < 0.001, ****p < 0.0001

Immediately prior to co-culture with EOC2 microglial cells, cortical cells were mechanically injured. Injured cortical cells were cultured for 2 DIV with or without EOC2 microglia suspended on 0.40 µm Transwell® inserts (Fig. 2). The site of injury (dashed white line) was observable and few neurofilament + (NF +) cells were found beyond the injury site without microglia (Fig. 2a, b). In cortical cultures with microglia, the site of injury was associated with increased cell density and increased NF expression at the site of injury (Fig. 2c, d). EOC2 microglial cells used for co-culture experiments are CD11b + (Fig. 2e, f).

Cortical cell viability following injury and co-culture with microglia was measured using 3-(4,5-Dimethylthiazol-2-yl)-2,5-Diphenyltetrazolium Bromide (MTT) colorimetric assays and chemiluminescent CellTitre-Glo® assays (CTG) that measure metabolic activity in living cells. Uninjured cortical cultures with and without EOC2 microglial cells were also assessed using the MTT and CTG assays. In the absence of cortical cell injury, microglial-derived soluble cues significantly enhanced cortical cell viability to 151.5 ± 13.6%, **p < 0.01, n = 3) that of uninjured cortical cells cultured in media alone (Fig. 2g). Co-culture of injured cortical cells with microglia significantly increased viability to 185.3 ± 17.1%. Increase viability of ~ 34% in injured cortical cells co-cultured with microglia as compared to uninjured cortical cells co-cultured with microglia was significant (*p < 0.05, n = 3). Viability of injured cortical cells alone was not significantly different from MTT activity in uninjured cortical cell controls (Fig. 2g, p > 0.05, n = 3). CTG chemiluminescent cell viability assays demonstrated increased significant differences in vortical cell viability between treatment groups (Fig. 2h). Co-culture with microglia enhanced uninjured cortical cell viability to 171.2 ± 1.0% and injured cortical cell viability to 207.7 ± 2.2% that of uninjured cortical cells in media alone (Fig. 2h, ****p < 0.0001, n = 3). The increase in viability of injured cortical cells co-cultured with microglia of ~ 36% was significant (Fig. 2h, **p > 0.01).

**Fig. 3:**
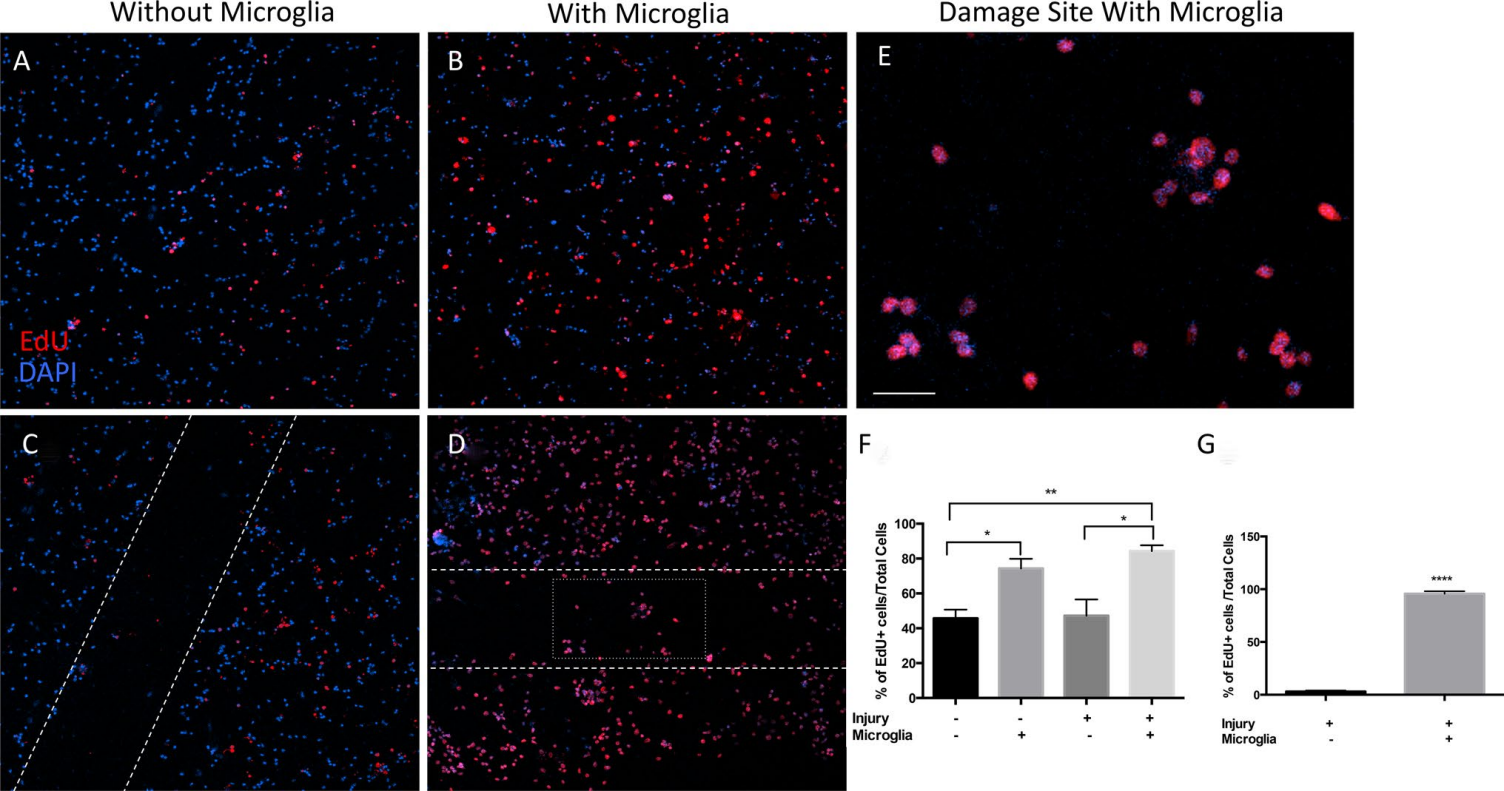
EOC2 microglial co-culture increased proliferation of uninjured and injured cortical cells. **a** Click-iT® EdU Alexa Fluor 647 (red) immunofluorescent staining of uninjured cortical cells cultured in the absence of microglia. **b** Click-iT® EdU Alexa Fluor 647 immunofluorescent staining of uninjured cortical cells co-cultured with microglia. In the presence of microglial co-culture, EdU + cells increased. **c** Click-iT® EdU Alexa Fluor 647 immunofluorescent staining of injured cortical cells cultured in the absence of microglia. Injury is indicated by the dashed white line. **d** Click-iT® EdU Alexa Fluor 647 immunofluorescent staining of injured cortical cells co-cultured with microglia. Injury is indicated by the dashed white line. The site of injury shown in **e** is indicated by the dashed white rectangle. **e** Full magnification of the injury site (dashed rectangle in **d** showing EdU + cells within the site of injury. Hoechst immunofluorescence (blue) indicates nuclei **a**–**e**. All images were taken with the Leica confocal using the 20 X objective. Scale bar represented 100 µm and applies to **a**–**d** where **e** shows full magnification and partial view of the imaging field. **f**. Quantification of EdU + primary cortical cells. The percent of EdU + cells in each experiment was calculated from the total number of cells counted. Cells were identified by using Hoechst to immunostain nuclei (Blue). In three experiments, 300 or more cells in each field were counted for each condition. Error bars represent Standard Deviation (SD). One-way ANOVA followed by Tukey’s multiple comparisons test was performed to determine the significance and a comparison of treatment groups yielded a F value = 9.804. Significance is *p < 0.05, **p < 0.01. **g** Quantification of EdU + cells at the site of injury. The percent of EdU + cells in injured cortical cell cultures with and without microglia was determined by counting a total of at least 50 cells in 6 areas of injury. The percent of Edu + cells in the total population of counted cells was determined for three separate experiments. Error bars represent standard deviation (SD). Significance (****p < 0.0001) was determined using a two-tailed unpaired T test. (Color figure online)

To examine cell proliferation in this co-culture system, incorporation of a modified, fluorescently labeled thymidine analogue EdU into newly synthesized DNA of proliferating cells was measured. Large field confocal image analysis of uninjured cortical cells without microglia demonstrating that these cultures include dividing cells at 2 DIV (Fig. 3a). In the presence of microglia, EdU + cells increased in uninjured cortical cell culture (Fig. 3b). Mechanical injury stripped away cortical cells from the culture surface as indicated by dashed white lines (Fig. 3c, d). Without microglia, few EdU + cells were observed in the injury site (Fig. 3c). With microglia suspended above injured cortical cells, an increase in proliferating EdU + cells was seen throughout the culture and within the injured area (Fig. 3d). Full magnification of the boxed area within the injured site (Fig. 3d) is shown in Fig. 3e. Quantification of the percent of proliferating cells in uninjured cortical culture without microglia showed that 45.7 ± 5.0% of the cells were EdU + (Fig. 3f). In the presence of microglia, the average percent of EdU + cells increased to 74.3 ± 5.6%. This 28.6 ± 7.5% increase in EdU + cells in the presence of microglia was significant (Fig. 3f, *p < 0.05, n = 3). Following injury, the percent of EdU + cells in cortical cultures without microglia was 47.2 ± 9.3% and was not significantly different from control, uninjured cortical cells cultured without microglia (Fig. 3f, p > 0.05, ± represents SD, n = 3). When cultured with microglia, the percent of proliferating EdU + cells in injured cortical cultures of 84.3 ± 3.3% was significantly different from the percent of EdU + cells in uninjured control conditions (Fig. 3f, **p < 0.01, n = 3) and from the percent of EdU + cells in injured conditions without microglia (Fig. 3f, *p < 0.05, n = 3). The difference in the percent of proliferating cells between uninjured and injured cortical cells co-cultured with microglia did not reach significance using quantification of injured and uninjured areas (Fig. 3f, p > 0.05, n = 3). Interestingly, significant differences are observed in Edu + cells at sites of injury in co-cultures with and without microglia (Fig. 3g). In the presence of microglia, ~ 95% of cells in the injured area EdU + while without microglia ~ 5% of the cells are EdU + (****p < 0.0001, Fig. 3g).

**Fig. 4:**
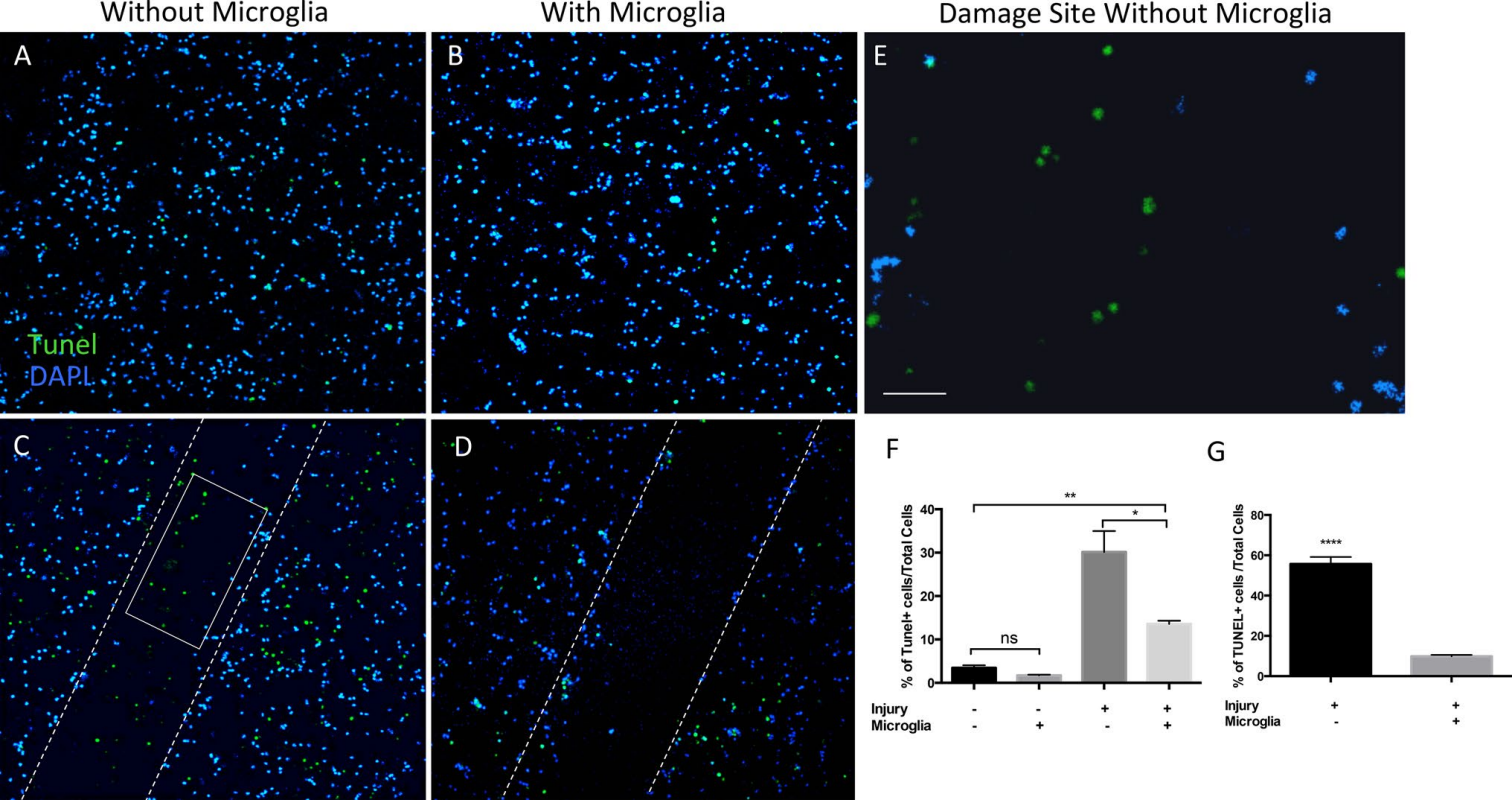
EOC2 microglial co-culture reduced cell death of injured cortical cells. **a** Click-iT® TUNEL Alexa Fluor 488 (green) immunofluorescent staining of uninjured cortical cells cultured in the absence of microglia. **b** Click-iT® TUNEL Alexa Fluor 488 immunofluorescent staining of uninjured cortical cells co-cultured with microglia. **c** Click-iT® TUNEL Alexa Fluor 488 immunofluorescent staining of injured cortical cells cultured in the absence of microglia. At the site of injury and beyond, TUNEL + cells were present. **d** Click-iT® TUNEL Alexa Fluor 488 immunofluorescent staining of injured cortical cells co-cultured with microglia. A reduction of TUNEL + cells at the site of injury and beyond was noticeable and significant **f**. Hoechst immunofluorescence (blue) indicates nuclei **a**–**d** All images were taken with the Leica confocal using the 20 X objective. Scale bar represented 100 µm and applies to **a**-**d** where **d** shows full magnification and partial view of the imaging field. **f** Quantification of TUNEL + primary cortical cells. The percent of TUNEL + cells in each experiment was calculated from the total number of cells counted. Cells were identified by using Hoechst to immunostain nuclei (Blue). In three experiments, 300 or more cells in each field were counted for each condition for quantification. Error bars represent standard deviation (SD). One-way ANOVA followed by Tukey’s multiple comparisons test of treatment groups yielded a F value = 29.35. Significance is *p < 0.05, **p < 0.01, ns is not significant. **h** Quantification of TUNEL + cells at the site of injury. The percent of TUNEL + cells in injured cortical cell cultures with and without microglia was determined by counting a total of at least 50 cells in 6 areas of injury. Error bars represent standard deviation (SD). Significance (****p < 0.0001) was determined using a two-tailed unpaired T test. (Color figure online)

To evaluate the effect of EOC2 microglial cells on cell survival, Click-iT® fluorescent terminal deoxynucleotidyl transferase dUTP nick end labeling (TUNEL) assays were performed (Fig. 4). In the absence of microglia, very few TUNEL + cells were present in uninjured cortical cell cultures (Fig. 4a). Following injury, cortical cells in the absence of microglia, showed increased TUNEL expression particularly in the area of injury (Fig. 4c). When injured cortical cells were co-cultured with microglia, a decrease in TUNEL + cells was observed both within the injured area and throughout the cell culture (Fig. 4d). Full magnification of the boxed area within the injury site clearly revealed the presence of TUNEL + cells (Fig. 4e). Quantification of TUNEL staining in uninjured cortical cultures without microglia shows that 1.72 ± 0.2% of the cells were TUNEL + . Co-culture of microglia with uninjured cortical cells did not significantly alter the percent of TUNEL + cells (3.44 ± 0.6%, n = 3, p > 0.05, Fig. 4f). Following injury, the percent of TUNEL + cells in cortical cultures without microglia significantly increased to 30.1 ± 4.9% (n = 3, *p < 0.05, Fig. 4f). When cultured with microglia, the number of TUNEL + cells in injured cortical cultures decreased to 5.6 ± 1.2% ( n = 3, Fig. 4f). The reduction of TUNEL staining by 24.6 ± 4.8% in injured cortical cells co-cultured with microglia was highly significant (**p < 0.01, n = 3, Fig. 4f). The percent of TUNEL + cells in uninjured cortical cultures in the presence of EOC2 microglial cells was not significantly different from the percent of TUNEL + cells observed in uninjured cortical cultures used as control (p > 0.05, n = 3, Fig. 4f). When looking specifically at the site of injury significant differences in TUNEL + cells were observed between injured cortical cells co-cultured with microglia and those cultured alone. In the absence of microglia, ~ 55% of cells in injured areas were TUNEL + while in the presence of microglia ~ 10% were TUNEL + (****p < 0.0001, Fig. 4g).

**Fig. 5:**
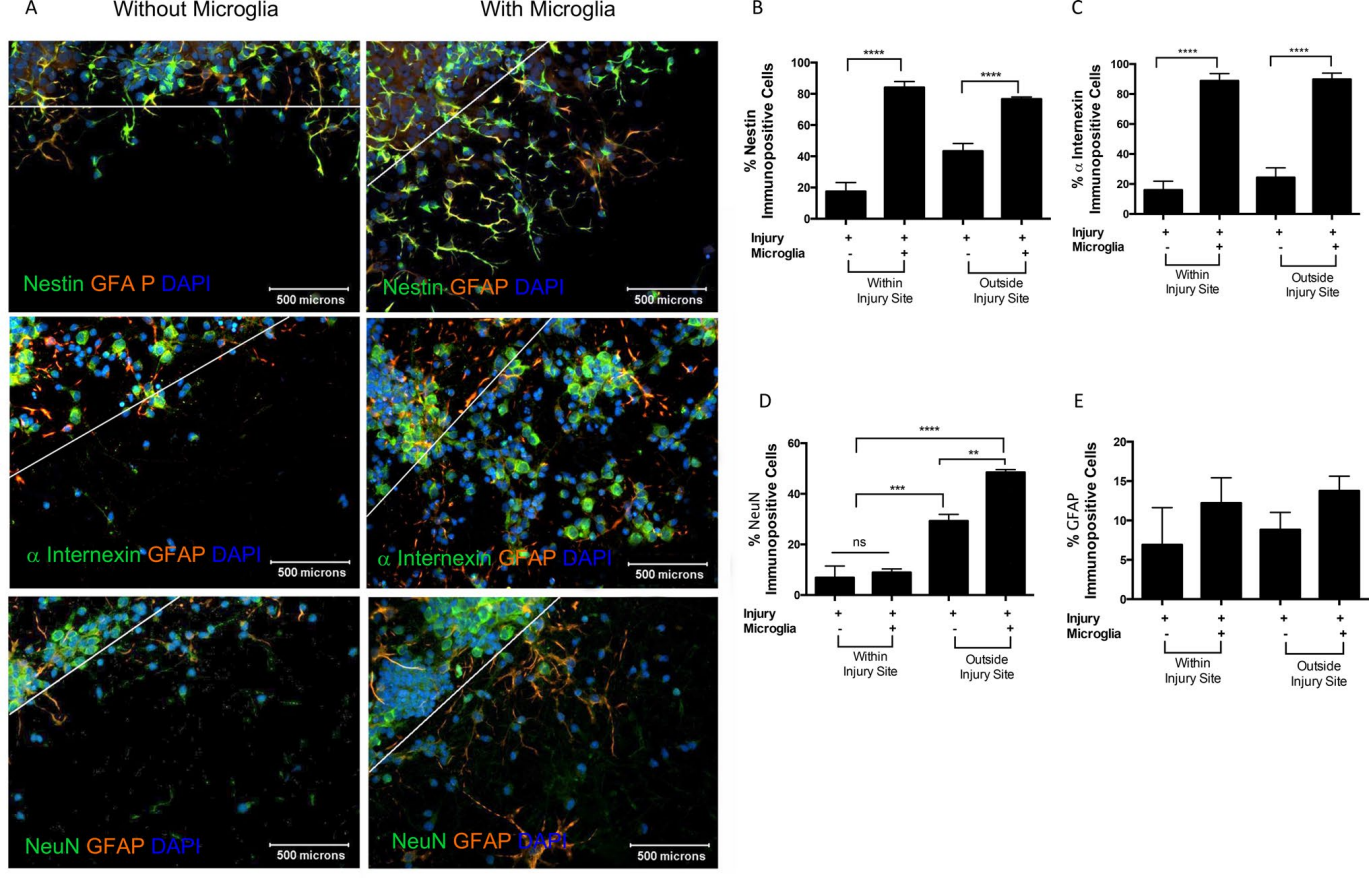
EOC2 microglia increase the percent of cortical cells expressing neurogenic markers, particularly within the site of injury. The percent of cells Nestin + (**a**), GFAP + (**b**), α-internexin + (**c**), and NeuN + (**d**) immunopositive cells was determined by comparing the number of immunopositive cells to all cells that were counted in each culture condition. Cells were identified by using Hoechst to immunostain nuclei. Cortical cells cultured alone served as control. Uninjured and injured cortical cells were cultured alone or in the presence of EOC2 microglia suspended on Transwells®. Quantification of immunocytochemical data within the injury site and outside the injury site is shown. Error bars represent SEM. One-way ANOVA followed by Tukey’s multiple comparisons test was performed to determine the significance and a comparison of treatment groups yielded a F value = 11.10. Significance is *p < 0.05, **p < 0.01, ***p < 0.001, *****p < 0.0001, ns is not significant. (Color figure online)

At the site of injury, microglia soluble cues significantly increase proliferation of cells and reduce cell death (Figs. 3 and 4). Such cell proliferation may involve neurogenesis. Progressive expression of proteins such as Nestin, GFAP, α-internexin, and NeuN is indicative of stages of neurogenesis [27, 58, 61]. Increased neurogenic protein expression in cortical and microglial co-cultures at the site of injury is of interest. Microglial soluble cues increased Nestin (green), GFAP (red), Nestin/GFAP cells (yellow), and α-internexin (green), and to a lesser extent NeuN (green) immunofluorescence within and outside of the site of injury (Fig. 5a). Microglia increased the percent of Nestin + cells within the site of injury by more than 45% and by ~ 38% outside the site of injury as compared to control, cortical cultures without microglia. These differences were highly significant (Fig. 5b, ****p < 0.0001). Within the site of injury, cortical cells co-cultured without microglia contained ~ 20% Nestin + cells while outside the injury site ~ 43% were Nestin + (Fig. 5a, b). Co-culture with microglia significantly increased Nestin + cells at the site of injury to 84.0 ± 3.8% and outside the site of injury to 76.6.0 ± 1.3% (Fig. 5b, ****p < 0.0001). Injury of cortical cultures in the absence of microglia resulted in ~ 15% of the cells being α-internexin + at the site of injury and ~ 24% of the cells being α-internexin + outside the site of injury (Fig. 5c, p > 0.05). Co-culture of injured cortical cells with microglial-derived soluble cues significantly increased α-internexin + cells by ~ 73% within the site of injury to 88.8 ± 4.3% and over 74% outside the site of injury to 89.8 ± 4.1% (Fig. 5c, ****p < 0.0001). Approximately 20% of cortical cells were NeuN + at the site of injury when cultured with or without microglia (Fig. 5d). Outside the site of injury, the percent NeuN + cells in cultures without microglia was 29.3 ± 2.6% and 48.5 ± 1.1% (Fig. 5d). Higher power images of NeuN immunoreactivity show NeuN + cells at the site of injury and outside the side of injury (Online Resource 1). An increase in the number of total cells was seen in cortical cultures in the presence of EOC2 microglial-derived soluble cues as compared to cortical cultures alone (Online Resource 1a, b). The percent of GFAP + cells with star-like extending processes in uninjured and injured cortical cultures with microglia was not significantly different from that seen in cortical cultures without microglia largely because of the low number of GFAP + cells and variability in the prevalence of GFAP + cells throughout the culture environment (Fig. 5a, e, p > 0.05).

**Fig. 6:**
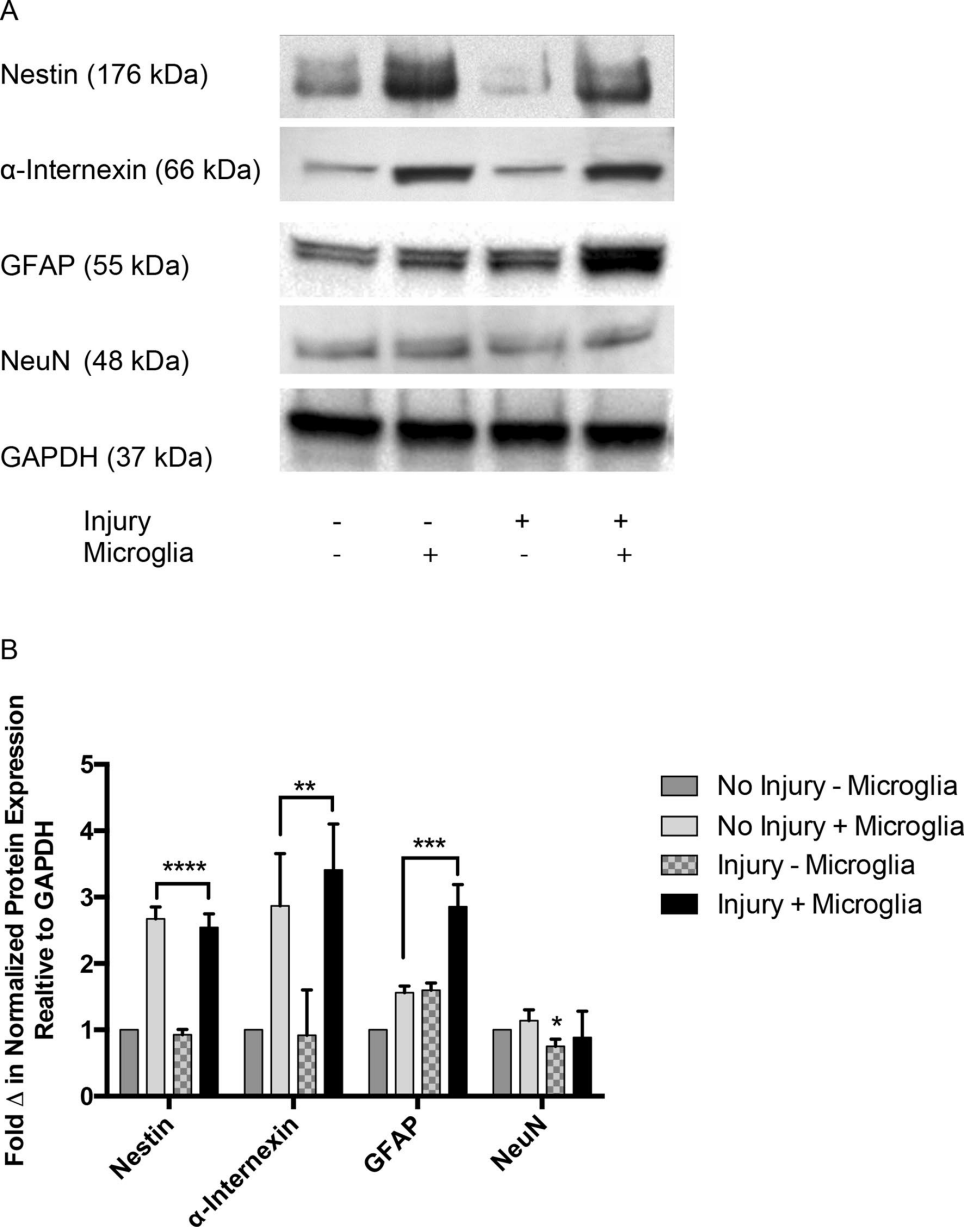
Western blot analysis of Nestin, α-internexin, NeuN, and GFAP expression in uninjured and injured cortical cultured exposed to EOC2 microglia or control media. **a** Representative western blot images of protein from uninjured cortical cultures in control media without microglia, uninjured cortical cultures with microglia, injured cortical cultures in control media without microglia, and injured cortical cultures with microglia. GAPDH was used as a total protein loading control. **b** Quantification of relative protein expression in western blot experiments. Experiments were run in triplicate using primary cultures from three biological replicates. Error bars represent SEM. Two-way ANOVA followed by Tukey’s multiple comparisons test was performed to determine the significance and yielded a F value = 50.44. Significance is *p < 0.05, **p < 0.01,***p < 0.001, *****p < 0.0001

Western blot analysis was used to assess protein expression of neurogenic markers in injured as well as uninjured cortical cultures (Fig. 6a). Nestin protein in uninjured and injured cortical cultures increased 1.7 ± 0.1fold and 1.5 ± 0.1fold respectively following co-culture with EOC2 microglia as compared to Nestin in uninjured control cultures alone (****p < 0.0001, ***p < 0.001, Fig. 6b). A 1.9 ± 0.5fold increase in α-internexin was observed in uninjured cortical cultures co-cultured with microglia as compared to control, uninjured neurons cultured alone (**p < 0.01, Fig. 6b). Injured cortical cells co-cultured with microglia exhibited a 2.4 ± 0.4fold increase in α-internexin compared to control, uninjured neurons cultured without microglia (**p < 0.01, Fig. 6b). GFAP increased 1.6 ± 0.1fold in uninjured cortical cells co-cultured with microglia and 1.9 ± 0.2fold in injured cortical cells co-cultured with microglia as compared to control uninjured cortical cells (***p < 0.001, Fig. 6b). GFAP also significantly increased ~ 1.6 fold in injured cortical cells without microglial co-culture (***p < 0.001, Fig. 6b). Injured cortical cells alone showed a 0.25 ± 0.1fold decrease in NeuN as compared to uninjured control cortical cultures (*p < 0.05, Fig. 6b). NeuN protein expression in injured cortical cells co-cultured with microglia compared to that in control conditions did not reach significance (p > 0.05, Fig. 6b). Entire western blot images are presented in Online Resource 2. BV2 microglial cell lines co-cultured with injured cortical cells appear to similarly increase Nestin and α-internexin expression as shown in Online Resource 3.

**Fig. 7:**
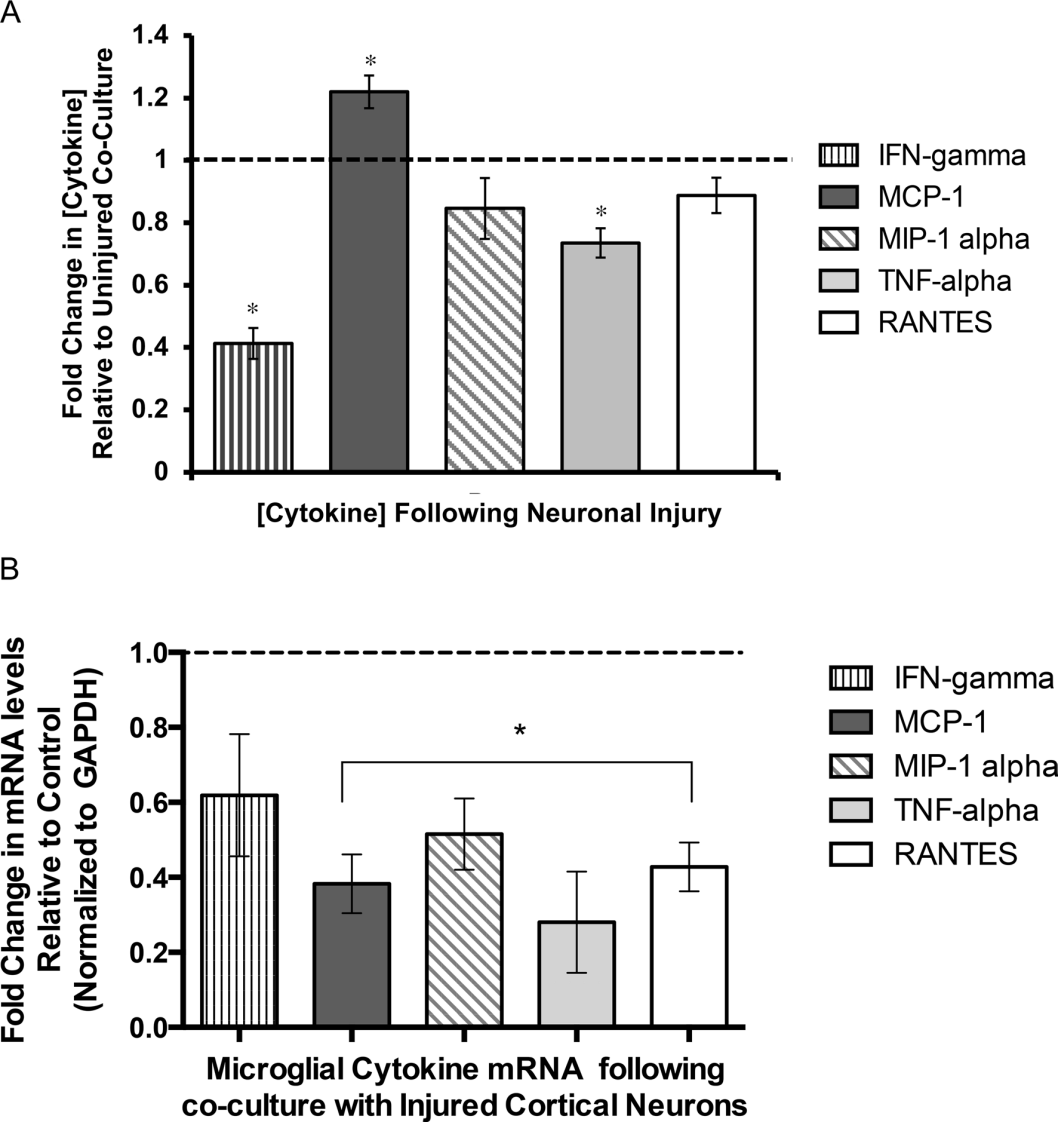
Multiplex ELISA and RT-PCR analyses of inflammatory cytokine protein and mRNA following co-culture with uninjured or injured cortical cells. **a** Relative cytokine levels in media collected from injured cortical cell and microglial co-culture as measured by multiplex ELISA assays are shown. The dashed line represents the normalized cytokine levels for IFN-γ, MCP-1, MIP-1α, TNF-α, and RANTES in media collected from co-cultures of EOC2 microglia and uninjured cortical cells. Normalized uninjured co-culture cytokine concentrations were set equal to one. The experimental data represent the average fold change in each cytokine as measured in the media collected from injured cortical cell and EOC2 microglial co-culture. Experiments were run in triplicate from three biological replicates. Error bars represent SEM. Students T Test was used to determine whether the fold change was significance as compared to normalized control, Significance is *p < 0.05. **b** qRT-PCR analysis of cytokine mRNA levels in EOC2 microglia following stimulation with injured cortical cells. EOC2 mRNA was collected from microglia suspended above cortical cultures on Transwells®. Fold change in mRNA levels was normalized to Gapdh expression in EOC2 microglia following stimulation with injured cortical cells. Fold change is compared to mRNA in EOC2 microglia co-cultured with uninjured cortical cells. Control mRNA expression is indicated by the dashed line set at one. MIQE guidelines were followed. Mouse specific primers were used for qRT-PCR analysis of mouse microglial cells. Experiments were run in triplicate for three biological replicates. Error bars represent SEM. Significance was determined using BioRad CFX Manager software. Significance *p < 0.05 was determined using a two-tailed unpaired T test

In order to begin to examine changes in the cytokine environment of in response to injury, multiplex ELISA assays were used to determine the presence of well-characterized cytokines in microglial-conditioned media. Media collected from co-cultures of injured cortical cells and EOC2 microglia was compared to media collected from co-cultures of uninjured cortical cells and EOC2 microglia. Analysis of three separate assays performed in triplicate showed that the concentration of several cytokines was significantly different from the levels observed in microglial-conditioned media collected from uninjured cortical cells that serves as the control (Fig. 7a). Monocyte chemoattractant protein-1 (MCP-1) concentration increased 22.0 ± 0.02% above control levels while IFN-γ and TNF-α concentration decreased to 41.3 ± 0.07% and 73.5 ± 0.08% below control levels in media collected from injured cortical cell and microglial co-culture compared to media from uninjured cortical cell and microglial co-culture, respectively (*p < 0.05, Fig. 7a). Concentrations of MIP-1α and RANTES decreased by ~ 20% in media from injured cortical and microglial co-cultures compared to media from control uninjured cortical and microglial co-cultures but these decreases were not significant (p > 0.05, Fig. 7a). IL-1α, IL-1β, IL-2, IL-4, IL-6, and GM-CSF were either undetectable or not significantly different in conditioned media from uninjured and injured cortical and microglial co-cultures (Online Resource 4). To begin to investigate whether the significant differences in cytokine concentrations detected in our microglial-conditioned medias are due to changes in microglial mRNA levels for these cytokines, RT-PCR was used to compare mRNA levels in EOC2 microglia following co-culture with injured or uninjured cortical cells. Since microglia are physically separated from cortical cells, microglial RNA can be specifically isolated. Decreased mRNA expression for IFN-γ by 22.2 ± 10.2%, MCP-1 by 79.7 ± 2.9%, MIP-1α by 60.2 ± 6.7%, TNF-α 97.6 ± 4.1%, and RANTES 62.5 ± 11.6% was observed in microglial co-cultured with injured cortical cells as compared to uninjured cortical cells (± is SEM, Fig. 7b). The dashed line represented the normalized mRNA levels in microglia cultured with uninjured cortical cells. Decreased expression of MCP-1 mRNA in EOC2 microglia suggests that the increase in MCP-1 protein levels was not microglial derived (Fig. 7b).

**Fig. 8:**
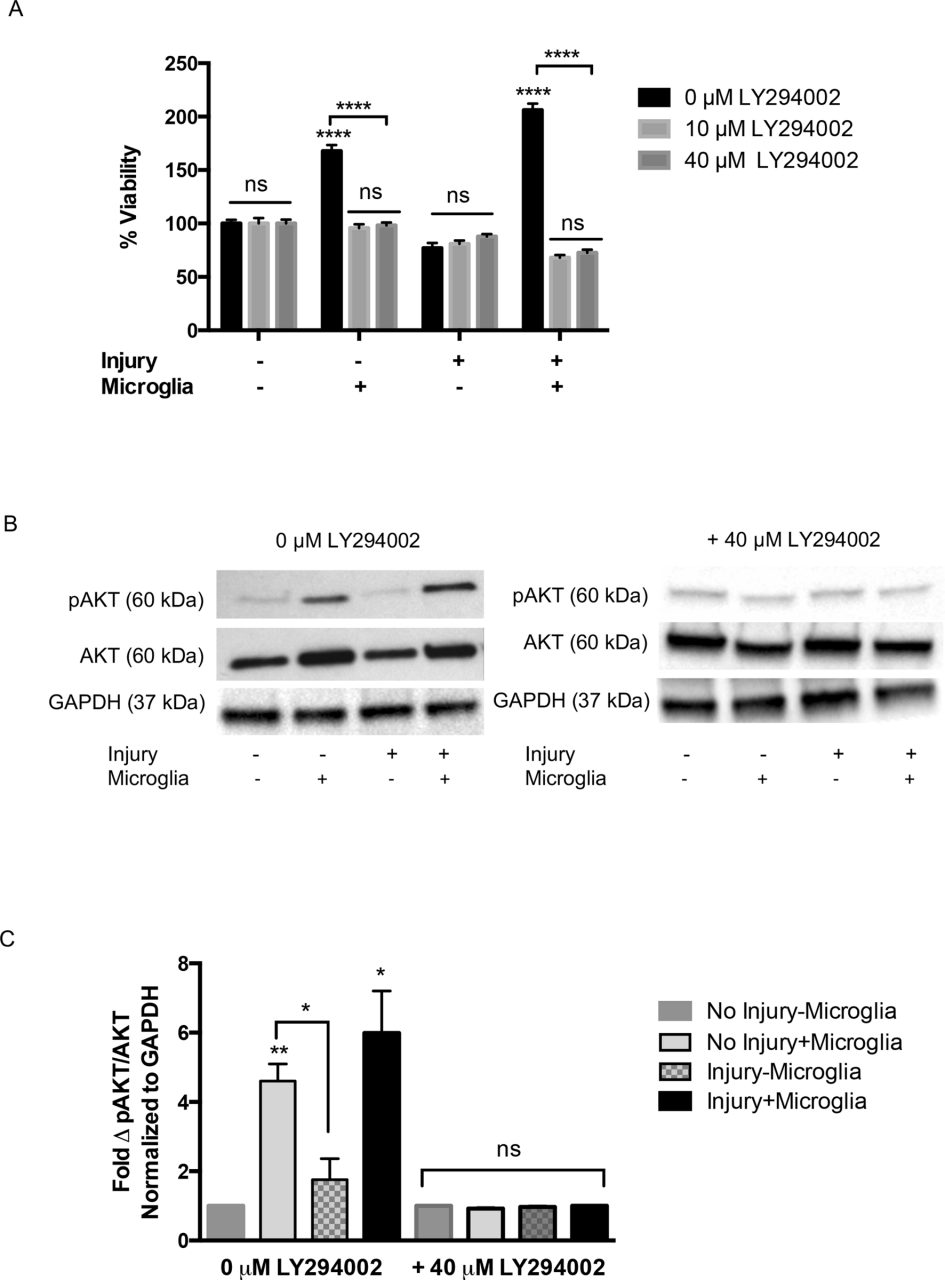
Effect of PI3K inhibition microglial-enhanced cortical cell viability and AKT phosphorylation in cortical cells following EOC2 microglial co-culture. **a** Quantification of MTT viability following LY294002 treatment of uninjured and injured cortical cells alone and in microglial co-culture. For each concentration, two-way ANOVA was used to determine significance effect of the inhibitor. Multiple comparisons yielded a F value for inhibitor interactions = 235.7 and F value between treatments = 146.6. Significance is ****p < 0.0001, ns indicates not significant. **b** Representative western blots illustrating pAKT phosphorylation in injured and injured cortical cultures with and without microglial co-culture. Culture conditions treated with 0 µM and 40 µM are shown. **c** Quantification of AKT phosphorylation as compared to total AKT protein levels normalized to GAPDH. Three separate western blot experiments were analyzed, data were averaged and error bars represent SEM. Two-way ANOVA was used to compare the significance the data for 0 µM and 40 µM LY294002 treatments. Multiple comparisons yielded a F value = 485.8. Significance is *p < 0.05, ns is not significant

Several signaling pathways are activated by soluble signaling molecules important for neurogenesis [6] and may also underlie the microglial-enhanced neurogenic protein expression observed in our co-culture system. To begin to investigate possible signaling pathways important for microglial-enhanced neurogenic protein expression, injured and uninjured cortical cells in co-culture with and without EOC2 microglia were treated with inhibitors for intracellular signaling pathways. We used viability assays to screen for those inhibitors that blocked microglial-enhanced viability of cortical cells. Microglial co-culture increased viability of uninjured and injured cortical cultures as compared to cortical cultures alone as shown previously (***p < 0.001, Online Resource 5). Inhibitors for MEK (PD98059), p38 MAPK (SKF86002), PKCα/βI/βII/γ (GF109203X), and Janus Kinase 2 protein (AG490) did not block the increased metabolic activity and viability of cortical cells co-cultured with microglia. AG490 and GF109203X at 40 µM did significantly influence viability of cortical cultures but these effects were not specific for microglial-enhanced viability (Online Resource 5). LY294002, an inhibitor of PI3K specifically reduced microglial-enhanced cortical cell viability of 137.7 ± 29.9% to 86.2 ± 7.3% and 89.7 ± 6.2% at 10 and 40 µM respectively (****p < 0.0001, Fig. 8a). Following injury, 10 and 40 µM LY294002 treatment significantly reduced microglial-enhanced cortical cell viability of 206.0 ± 6.1% to 67.9 ± 2.6% and 72.7 ± 2.7% respectively (****p < 0.0001, Fig. 8a). Treatment of uninjured or injured cortical cells in the absence of microglia with LY294002 did not significantly affect metabolic activity (p > 0.05, Fig. 8a). Analysis of western blots showed that microglia in uninjured cortical cultures increased AKT phosphorylation 3.6 ± 1.0fold as compared to uninjured cortical cells alone (*p < 0.05, Fig. 8b). Complete AKT western blot images are provided (Online Resource 6). Injury alone did not significantly increase (~ 0.8 fold) phosphorylation of AKT as compared to control levels (Fig. 8b, p > 0.05). Following injury and co-culture with microglia, AKT phosphorylation increased 5.0 ± 1.0fold compared to injured cortical cells alone (**p < 0.01, Fig. 8b). This increase was also significantly different from AKT phosphorylation measured in injured cortical cells without microglial co-culture (4.2 ± 1.0fold increase, *p < 0.05, Fig. 8b, d). LY294002 blocked the increase in AKT phosphorylation seen in cortical cells when cultured with microglia (Fig. 8c, d). A low (10 µM) and high (40 µM) concentration of LY294002 was selected based on concentrations used commonly in the published literature for cortical cultures in vitro and neither concentration alone negatively affected cortical cell viability [34, 42, 91].

**Fig. 9:**
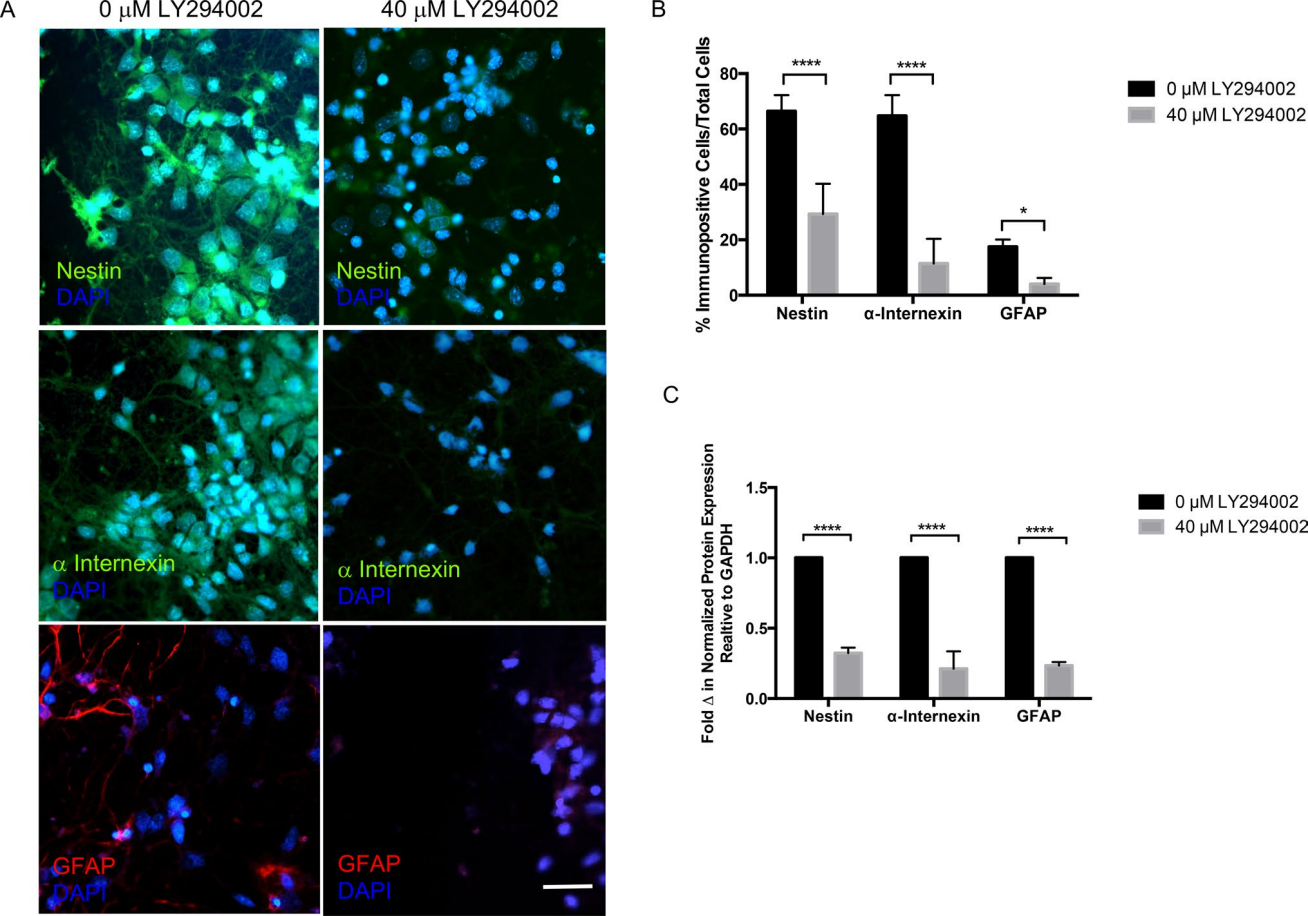
Inhibition of PI3K blocks microglial-enhanced expression of neurogenic markers in injured cortical co-cultures. **a** Immunofluorescence of Nestin (green), α-internexin (green), or GFAP (red) and DAPI (blue to indicate nuclei) in injured cortical cells co-cultured with microglia in 0 µM or 40 µM LY294002. Application of 40 µM LY294002 significantly reduced Nestin, α-internexin, and GFAP immunofluorescence. DAPI (blue) was used to observe nuclei of all cultured cells. All images were acquired with a 40X Leica objective. Scale bar represents 50 µm. **b** Quantification of immunofluorescence for neurogenic markers. Three separate fields within uninjured and injured cortical cultures that were treated with 0 µM or 40 µM LY294002 and stained for each neurogenic marker were evaluated for the number of Nestin + , α-internexin + , and GFAP + cells in the total number counted. At least 100 cells were counted per field. Error bars represent SEM. **c** Western blot analysis of Nestin, α-internexin, and GFAP expression in co-cultures with 40 µM LY294002 treatment were compared to control co-culture conditions. Experiments were performed in triplicate and the change in protein expression was normalized to the untreated controls for each protein. Error bars represent SEM. **b** and **c** Student’s t-test were used to determine the significance of LY294002 treatment in each condition and for each neurogenic marker. Significance is *p < 0.05, ****0.0001

Immunocytochemical analysis and western blot was used to evaluate the effect of blocking PI3K activity and AKT phosphorylation in the injured cortical cell-microglial co-cultures (Fig. 9). Co-cultures were established as previously described and cortical cells were pre-incubated with LY294002, then evaluated for expression of Nestin, α-internexin, and GFAP (Fig. 9a). Immunocytochemical analysis supported western blot data showing that LY294002 reduced Nestin, α-internexin, and GFAP expression in injured cortical cells co-cultured with microglia (Fig. 9a). Application of 40 μM LY294002 reduced the percent of Nestin + cells in co-cultures of microglia and injured cortical cells by ~ 37% and α-internexin expression by ~ 53% (****p < 0.0001, Fig. 9b). In co-cultures of microglia and injured cortical cells, 40 µM LY294002 reduced GFAP expression in injured cortical cells by ~ 13% (*p < 0.05, Fig. 9b). Western blot analysis of Nestin, α-internexin, and GFAP expression in the presence of microglia was normalized and the effect of blocking the PI3K/AKT pathway with 40 µM LY294002 was assessed. Application of 40 µM LY294002 reduced expression of Nestin to 0.68 ± 0.04fold, α -internexin to 0.67 ± 0.04fold, and GFAP to 0.76 ± 0.04fold as compared to each control (Fig. 9c). These experiments imply that phosphorylation of AKT may be necessary for microglial-enhanced expression of specific neurogenic proteins in cortical cells.

## Discussion

This work shows the ability of EOC2 microglial cells to support the viability, proliferation, neurogenesis, and survival of primary cortical cells during homeostasis and following mechanical injury. Cortical microglial cells are a potential source of neurogenic signaling molecules during development and potentially following injury. Microglia have been shown to contribute to neuronal synapse development, survival, and neurogenesis in neurogenic niches of the CNS during development [68, 70, 75]. The role that microglia play in neurogenesis, gliogenesis, neuronal degeneration and regeneration are debated and may depend on the nature and duration of the injury and immune response [31]. Recent characterization of primary microglia shows that microglia are highly diverse and that subpopulations of functional microglia are present within specific brain regions and polarize in response to particular environmental cues [28, 76]. Because of the diversity of primary microglia, this study presents an in vitro model system using the EOC2 microglial cell line and primary cortical cells in co-culture to begin to examine the neurogenic potential of microglial soluble cues during cortical cell homeostasis and following cortical cell injury. While useful, the microglial cell line may bias our results and future experiments necessarily involve the use of carefully isolated and regionally specific primary microglia. Suspension of microglia on 0.40 µm Transwell® cell culture inserts directly above cortical cells allows for evaluation of how the cortical environment is influenced by microglial-derived soluble cues. The methods of isolating primary cortical cells utilized in this in vitro system have been well characterized [52] and our data show that these cells express primarily neurogenic and neuronal cell markers with evidence of less than 5% astrocytic protein expression and less than 2% microglial protein expression. Using this system where EOC2 microglia and primary cortical cells are not in direct contact with each other also allows for protein and RNA to be collected independently from either cell population. This reductionist in vitro system allows for preliminary questions to be asked about microglial neurogenic potential in the cortex and can be used for future studies with other microglial cell lines and primary microglial cells. These studies do not negate the role of microglia in other areas of the brain such as the cerebellum studied using similar in vitro methods [79] .

Results from experiments show that soluble cues from EOC2 microglial cells responding to primary cortical cells enhanced cortical cell viability and proliferation, while promoting the expression of neurogenic and mature neuronal markers in the primary cortical cultures. Homeostatic as well as microglia activated by injury promoted the viability of primary cortical cells. One important finding is that cortical viability was significantly enhanced and cell death was significantly reduced within the site of injury in co-cultures of microglia and injured cortical neurons compared to uninjured cortical neurons, and microglia. Without microglial co-culture, enhanced proliferation is not seen within the injury site in cortical cultures. We were unable to detect significant differences in EdU incorporation between the two microglial co-culture conditions outside the site of injury and globally. This may be due to the small areas of injury and overall increase in cell proliferation, the sensitivity of the EdU assay, or a lack of significant microglial responses. In the absence of injury, microglia in the subgranular and subventricular zones can influence the number of mature neurons that are generated by regulating neuronal stem cell and neuronal progenitor cell proliferation and differentiation throughout life [17, 20, 48, 72]. Depletion of microglia using a colony stimulating factor receptor antagonist was shown to disrupt the recruitment of basal progenitors to the cortex in embryonic mouse brain [1]. While in the adult brain, microglial depletion had no effect on the proliferation of neural stem cells, transient amplifying cells, and neuroblasts in the neurogenic niche [37]. Further investigation of mechanism (s) of microglial proliferative properties is clearly warranted and the response of primary microglia is a necessary next step.

Another important finding is that microglial co-culture reduced cortical cell death in injured and uninjured cortical cultures. This was particularly evident at the site of injury when cortical cells were co-cultured with microglia. Microglia may also act locally to stimulate survival of responsive cortical cells at noncanonical neurogenic regions. Microglia can influence cell death in neurogenic zones and this response is dependent upon the combination of proinflammatory cytokines, growth factors, and phagocytic activity of microglia [6, 25, 47, 87]. Future studies could use this model to investigate primary microglia responses at different developmental ages and/or isolated from different brain regions. Additionally experiments investigating the inhibition of apoptotic pathways specifically, such as the activation of caspase 3, would help to determine whether microglia block specific forms of cell death in cortical cells.

EOC2 microglial-derived soluble cues influence neurogenic protein expression in cortical cells in vitro. Increased neurofilament was observed at the site of injury with microglial co-culture. Neurofilament is expressed in neuronal progenitor cells and mature neurons and is associated with structural maturation of neurons and axonal function [36]. The percent of cortical cells expressing Nestin, α-internexin, GFAP, and NeuN and the relative amount of Nestin, α-internexin, GFAP, and NeuN protein expression were significantly higher in uninjured and injured cortical cultures with EOC2 microglia as compared to control cultures without microglia. Nestin expression alone or Nestin and GFAP expression are characteristic of early, primary neurogenic stem cells and progenitors [4, 45, 46, 86, 88]. Nestin + progenitors can eventually give rise to intermediate progenitors that produce immature neurons or neuron-committed progenitors [30, 43, 53]. Nestin and GFAP are also co-expressed by migratory astrocyte progenitors in the neurogenic niche of the subependymal zone following trauma [7, 18, 33, 58]. Microglial-derived soluble signals may help shape a local environment that can stimulate Nestin + and GFAP + cell proliferation important for generation of new neurons and glia following injury in the cortex. The variable expression of GFAP in our culture system may be the result of its expression pattern in neurogenic cells as well as mature astrocytes. Microglial soluble signals have been shown to stimulate astrocyte differentiation from progenitor cells [57, 72]. Expression of α-internexin, a Type IV neuronal intermediate filament protein, occurs during later stages of neuronal differentiation and axon development [36]. The increase in α-internexin + cells and protein concentration at the injury site suggests that microglial responses to injury creates an environment that supports early stages of neurogenesis. Microglia and their role in promoting neurogenesis after injury has recently been supported by evidence that neuroblasts can be recruited from the subgranular zone of hippocampus to sites of injury in the cortex by microglial-derived specific cues [56]. These data and that of this study suggest that microglia, at least at specific times following injury, function to promote the proliferation neuronal progenitors. NeuN is a marker for mature neurons [27]. Microglia supported NeuN expression in both uninjured and injured cortical cultures. Few NeuN + cells are present in injured areas. Maturation of neuronal progenitors takes up to one week in culture [61]. The outcome of increased Nestin, GFAP, and α-internexin expression will require longer culture conditions that 2 DIV as in these studies. Our supplemental data showing increased NeuN immunofluorescence in the area of injury following cortical co-culture with EOC2 microglial cells, suggests that microglial-enhanced neurogenesis and differentiation of mature neurons is occurring (Online Resources 1, 2). Additional, long-term experiments are underway to determine whether maturation of mature neurons results from effector microglial-enhanced neurogenesis. Taken together, these data suggest that microglial soluble signals released following co-culture with cortical cells during homeostasis and more so during activation by cortical injury promote the proliferation and survival of neurogenic cells.

Multiplex ELISA assays of microglial-conditioned media from co-culture experiments with uninjured and injured cortical cells revealed that the expression of several cytokines significantly changed following EOC2 microglia stimulation by neuronal injury. Specifically, multiplex ELISA data showed significant upregulation of MCP-1/CCL2 and downregulation of IFN-γ, MIP-1α, TNF-α and RANTES. Upregulation of MCP-1/CCL2 is interesting since MCP-1/CCL2 is associated with inflammation [50, 89, 90] as well as subventricular zone and neocortical neurogenesis and neurogenic migration [14]. MCP-1/CCL2 is expressed by microglia, neurons, neural stem cells and astrocytes [32, 67]. Our data suggest that the increase in MCP-1 protein is unlikely to be microglial-derived since MCP-1 mRNA levels are lower in EOC2 microglia responding to injury than in control microglia (Fig. 8b). MCP-1 could be secreted from the increased number of Nestin + cells. Pluripotent cells have been shown to secrete MCP-1 and may contribute to the activation of microglia [14]. Work focused on investigating the upregulation of MCP-1 and the expression using RT-PCR is currently underway. Decreased levels of IFN-γ in combination with other inflammatory cytokines following co-culture of microglia with injured neurons may also favor neurogenesis in cortical cultures. Inflammatory cytokines are known to act at specific concentrations and in certain combinations to regulate neurogenesis, gliogenesis and neuronal survival [2, 6]. TNF-α, while primarily associated with inflammatory responses associated with neurotoxicity [8, 77] has varied effects on neurogenesis and can stimulate neurons to secrete CCL2 [88, 89]. Most recent studies show that suppression of TNF-α enhances neurogenesis [12]. MIP-1α and RANTES have diverse roles in the CNS. Various studies have shown that MIP-1α, RANTES, and other ligands binding CCR5 receptors on neurons contribute to pro-inflammatory neurotoxicity [8, 77]. However, these ligands may also play an important role in the development and migration of neurons [44]. Microglia can also secrete growth factors and neurotrophins [13]. Neurotrophin release may be enhanced when microglia respond to cortical injury [64]. Other soluble signals such as prokineticins may contribute to microglial-enhanced neurogenesis presented in this in vitro system [39, 56, 92]. We suggest, as have others [19, 21, 69, 70] that microglial effects on neuronal survival, proliferation, and differentiation are largely dependent upon the composition of soluble signals that are released by microglia in response to stimulation. Continued investigations are underway to better dissect the complex milieu of neurogenic soluble signals released by microglia or other secretory cells in the presence of microglia.

Microglial-derived cues have been shown to activate both MAPK and PI3K/AKT pathways while stimulating neurogenesis of cultured progenitor cells and in neurogenic niches [23, 24, 83]. PI3K and MAPK pathways underly cortical cell proliferation, survival, and differentiation and disruption of these pathways can lead to degeneration [65]. Our results showed that AKT and MAPK phosphorylation increased in cortical cultures following exposure to EOC2 microglial-conditioned media. However, application of the PI3K/AKT inhibitor, LY294002, blocks microglial-enhanced neuronal survival and proliferation following injury (Fig. 8) and the expression of neurogenic markers. Inhibition of other intracellular signaling pathways associated with viability, proliferation, and neurogenesis such as MEK [22], p38MAPK [29], PKCα/βI/βII/γ [23, 24, 83] and Janus Kinase 2 protein [35, 57, 60] did not block the increased viability of cortical cells co-cultured with EOC2 microglia.

Taken together, our data provide an enticing view of the dynamic and multifunctional role of microglia in the cortex. Microglia responding to cortical cues may stimulate local neurogenesis and potential repair after injury. Downregulation of pro-inflammatory cytokine production by microglia could allow for increased proliferation, reduced cell death and increased neurogenesis. Experiments demonstrating that specific upregulated or downregulated microglial-derived cytokines or combinations of cytokines are both necessary and sufficient for neurogenesis via activation of AKT signaling remain to be performed. Neuronally derived cytokines may also contribute to microglial-enhanced neurogenesis and additional evaluation of neuronal transcription and translation should be evaluated further. Future experiments examining the effect of the addition or neutralization of individual or combinations of cytokines should be examined using the co-culture system. Our results suggest that the microglia enhance neurogenesis and promote neuronal survival by stimulating the PI3K/AKT signaling pathway in cortical cells. While other intracellular signaling pathways are likely also stimulated, the inhibition of the PI3K/AKT pathway and not other pathways previously implicated in neurogenesis blocked EOC2 microglial-enhanced neurogenesis. Further elucidation of the intracellular mechanisms regulating neurogenic function of microglia is essential for understanding the intrinsic neuroprotective role of immune activity in the CNS and may aid in the development of methodologies to promote such activity during neurodegenerative disease or following traumatic injury. The in vitro model system presented here provides an experimental tool to investigate the mechanisms of primary microglial responses to cortical injury in future work.

## Supplementary Information

Below is the link to the electronic supplementary material.

Supplementary file1 Online Resource 1 NeuN expression in cortical cells following injury and co-culture with EOC2 microglia. A) Quantification of NeuN+ cells as a percent of the total number of cortical cells. NeuN+ cells were counted in three separate experiments in control conditions, following injury and in co-culture with microglia. A total of at least 300 cells were counted and data are presented as mean with error bars representing SEM. One-way ANOVA was used to determine significance, *p<0.05, **p<0.01, ns is not significant. B) Representative immunofluorescent image of injured cortical cells stained with NeuN and DAPI (for nuclei) 2DIV following injury. C) Representative immunofluorescent image of injured cortical cells co-cultured with microglia and stained for NeuN and DAPI (for nuclei). B-C) The dashed white line indicates the site of injury. The scale bar represents 50 µm (PDF 1004 KB)

Supplementary file2 Online Resource 2 Entire western blot images for analysis in Fig 6. Western blot images of Nestin, α-internexin, NeuN, GFAP, and GAPDH used as protein loading control. Lanes are designated as L: ladder, Lane 1: Protein from uninjured cortical cells cultured without microglia., Lane 2: Protein from uninjured cortical cells cultured with microglia., Lane 3: Protein from injured cortical cells cultured without microglia., Lane 4: Protein from injured cortical cells cultured with microglia (PDF 43 KB)

Supplementary file3 Online Resource 3 BV2 microglia co-cultured with cortical cells enhances neurogenic protein expression A) Immunofluorescence of Nestin+ (red), α-internexin+ (green), NeuN+ (red) and GFAP+ (green) cells in injured cortical cells co-cultured without or with microglia. DAPI (blue) was used to observe nuclei of all cultured cells. Scale bar represents 500 µm. All images were acquired with a 20X Leica objective. B) Representative western blot images of protein from injured cortical cultures in control media without microglia (-) or injured cortical cultures with microglia. GAPDH was used as a total protein loading control. C) Quantification of relative fluorescence of neurogenic protein expression. Three separate fields within injured cortical cultures were evaluated for protein expression using immunofluorescent measurement software to determine the fluorescence intensity units for each protein marker. Averaged fluorescent intensity data from injured cortical cell cultures were normalized and set equal to 1 to determine relative fluorescent intensity units (RFU). Fold change in RFU in injured cortical cultures with microglia was determined and multiple Student T Tests were performed to determine significance. Error bars represent SEM. Significance is **p<0.01, ***p<0.001, ****p<0.0001 (PDF 159 KB)

Supplementary file4 Online Resource 4 Multiplex ELISA and RT-PCR analyses of inflammatory cytokine protein and mRNA following co-culture with uninjured or injured cortical cells. IL-1, IL-1, IL-2, IL-4, IL-3, IL-6, IL-10, IL-12, 1L-17 and GM-CSF were either undetectable in all culture conditions or were not significantly different when detected in co-cultures of uninjured and injured cortical cells with microglia. Error bars represented SEM. Two-way ANOVA followed by Tukey’s multiple comparisons test was performed to determine the significance (PDF 274 KB)

Supplementary file5 Online Resource 5 Inhibitors of MEK, p38, or PKC intracellular signaling pathways do not specifically inhibit EOC2 microglial-enhanced cortical cell viability. A-D) Quantification of MTT viability assays were performed in triplicate experiments using three biological replicates of primary cortical cells. Average OD 595 nm values were converted to percent viability and are shown with error bars representing SEM. Inhibitors were applied at 0, 10, and 40 µM. A) Quantification of viability of uninjured and injured cortical cells alone or in co-culture with microglia in the presence of MEK inhibitor PD98059. B) Quantification of viability of uninjured and injured cortical cells alone or in co-culture with microglia in the presence of p38 MAPK inhibitor SKF86002 C) Quantification of viability of uninjured and injured cortical cells alone or in co-culture with microglia in the presence of PKCα/βI/βII/γ inhibitor GF109203X. D) Quantification of viability of uninjured and injured cortical cells alone or in co-culture with microglia in the presence of Janus Kinase 2 inhibitor AG490. Two-way ANOVA was used to determine significance of the inhibitors and treatment groups. For PD98059 the F value between treatment groups was 16.96. For GF109203 the F value between treatment groups was 81.74. For SKF86002 the F value between treatment groups was 4.83. For AG490 the F value between treatment groups was 11.28. *p,0.05, **p<0.01, ***p<0.001, ****p<0.0001, # indicates that percent viability was significantly different from that of control, uninjured cortical cells alone, ns indicates not significant (PDF 1920 KB)

Supplementary file6 Online Resource 6 Western blot images of AKT and phosphorylated AKT (pAKT) shown in Fig 8. Lanes are designated as L: ladder, Lane 1: Protein from uninjured cortical cells cultured without microglia., Lane 2: Protein from uninjured cortical cells cultured with microglia., Lane 3: Protein from injured cortical cells cultured without microglia., Lane 4: Protein from injured cortical cells cultured with microglia (PDF 1802 KB)

## Data Availability

The datasets used and/or analyzed during the current study are available from the corresponding author on reasonable request.

## Abbreviations

AKT: Protein Kinase B
PI3K: Phosphatidylinositol 3-kinase
E: Embryonic
TUNEL: Terminal deoxynucleotidyl transferase dUTP nick end labeling
NeuN: Fox-3, Rbfox3, or Hexaribonucleotide Binding Protein-3
Rbfox3: Hexaribomucleotide binding protein 3
RT-PCR: Reverse transcriptase polymerase chain reaction
MCP-1: Monocyte chemoattractant protein 1
CCL2: Chemokine ligand 2 or monocyte chemotactic protein 1
IFN-,γ: Interferon gamma
MIP-1α: Macrophage inflammatory protein 1 alpha
TNFα: Tumor necrosis factor alpha
RANTES (CCL5): Regulation on activation, normal T cell expressed and secreted, chemokine ligand 5
CNS: Central nervous system,
BDNF: Brain derived neurotrophic factor
GDNF: Glial derived neurotrophic factor
STAT: Signal transducer and activation of transcription
NFκB: Nuclear factor kappa-light-chain-enhancer of activated B cells
TGF-β: Transforming growth factor beta
IGF-1: Insulin growth factor
MAPK: Mitogen activated protein kinase
CA1: Cornu Ammon 1 of the hippocampus
FGF: Fibroblast growth factor
EGF: Epidermal growth factor
IL1-13: Interleukins 1–13
GFAP: Glial fibrillary acidic protein
TUJI: Neuron specific class III beta tubulin
SEM: Standard error of the mean
NF: Neurofilament
MTT: 3-(4,5-Dimethylthiazol-2-yl)-2,5-Diphenyltetrazolium Bromide
O.D.: Optical density
EdU: 5′-Ethynyl-2′-deoxyridine
DNA: Deoxyribonucleic acid
RFU: Relative immunofluorescence units
DAPI: 4′,6-Diamino-2-phenylindole
DIV: Days in vitro
ELISA: Enzyme-linked immunosorbent assay
GM-CSF: Granulocyte macrophage colony stimulating factor
mRNA: Messenger ribonucleic acid
MEK: Mitogen-activated protein kinase kinase
p38 MAPK: P38 mitogen-activated protein kinase
PKC: Protein kinase C
DMEM: Dulbecco’s Modified Eagles Media
FBS: Fetal bovine serum
DMSO: Dimethylsulfoxide
PBS: Phosphate buffered saline
Gapdh: Glyceraldehyde 3-phosphate dehydrogenase
ΔΔCt: Delta-delta cycle threshold
CTG: CellTitre-Glo®
EdU: 5-Ethynyl-2′-deoxyuridine
TUNEL: Terminal deoxynucleotidyl transferase dUTP nick end labeling
