# Supplementary figures and images for: Microglia induce neurogenic protein expression in primary cortical cells by stimulating PI3K/AKT intracellular signaling in vitro
Source: Mol Biol Rep. 2021 Jan 2;48(1):563–84. doi: 10.1007/s11033-020-06092-0 (PMC7884585; doi:10.1007/s11033-020-06092-0)

NESTIN 176 kDa

1. 2. 3. 4. L

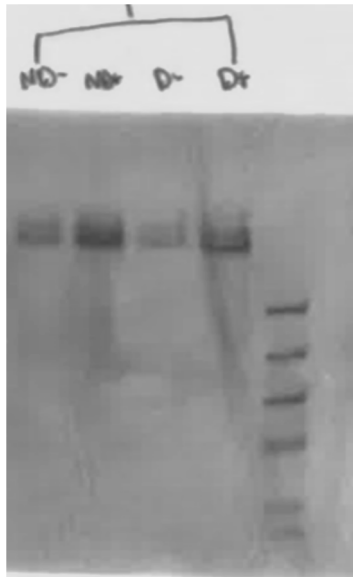

$\alpha$ -Internexin 66 kDa

1. 2. 3. 4. L

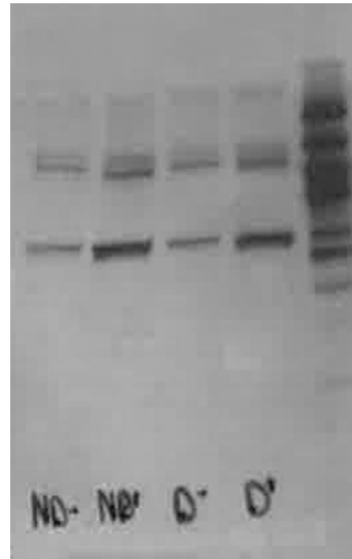

NeuN 48 kDa

BM 1. 2. 3. 4.

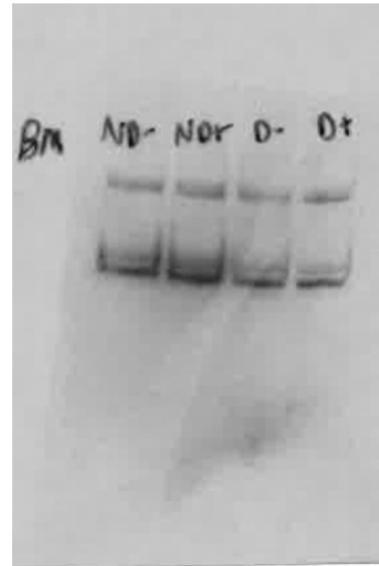

GFAP 55 kDa

1. 2. 3. 4. L

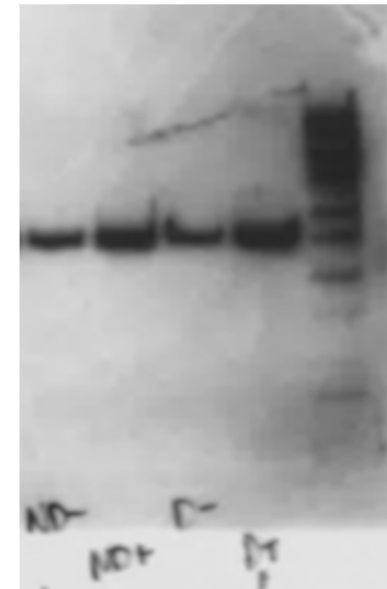

GAPDH 37 kDa

L 1. 2. 3. 4.

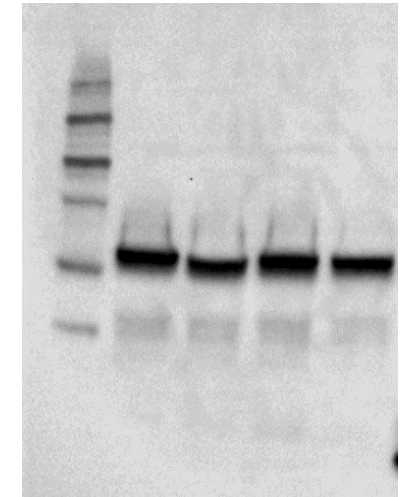

Supplement: Supplementary file 1 — Supplementary file1 Online Resource 1 NeuN expression in cortical cells following injury and co-culture with EOC2 microglia. A) Quantification of NeuN+ cells as a percent of the total number of cortical cells. NeuN+ cells were counted in three separate experiments in control conditions, following injury and in co-culture with microglia. A total of at least 300 cells were counted and data are presented as mean with error bars representing SEM. One-way ANOVA was used to determine significance, *p<0.05, **p<0.01, ns is not significant. B) Representative immunofluorescent image of injured cortical cells stained with NeuN and DAPI (for nuclei) 2DIV following injury. C) Representative immunofluorescent image of injured cortical cells co-cultured with microglia and stained for NeuN and DAPI (for nuclei). B-C) The dashed white line indicates the site of injury. The scale bar represents 50 µm (PDF 1004 KB) [file 11033_2020_6092_MOESM1_ESM.pdf]

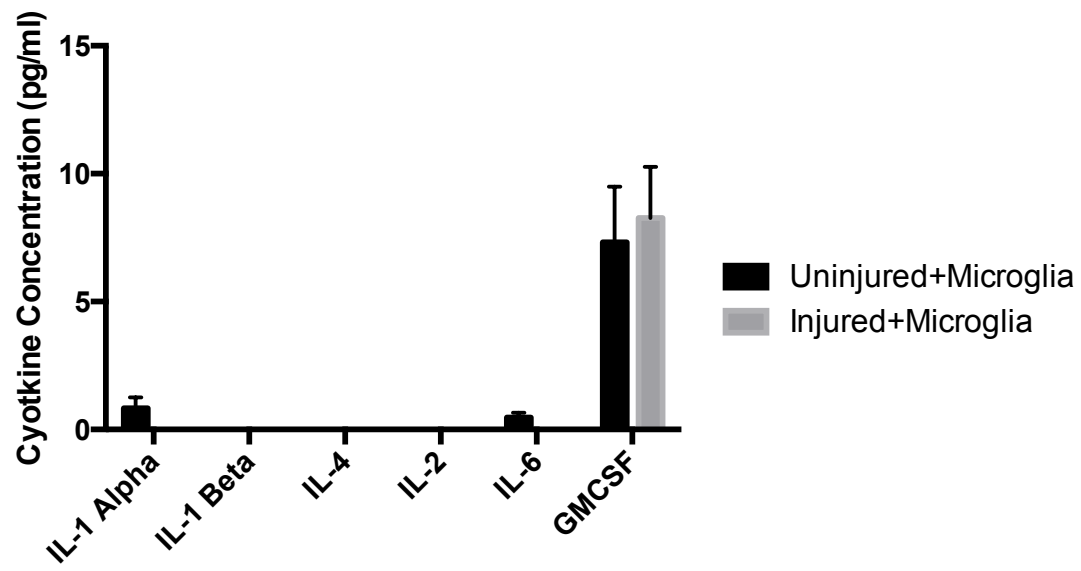

Supplement: Supplementary file 2 — Supplementary file2 Online Resource 2 Entire western blot images for analysis in Fig 6. Western blot images of Nestin, α-internexin, NeuN, GFAP, and GAPDH used as protein loading control. Lanes are designated as L: ladder, Lane 1: Protein from uninjured cortical cells cultured without microglia., Lane 2: Protein from uninjured cortical cells cultured with microglia., Lane 3: Protein from injured cortical cells cultured without microglia., Lane 4: Protein from injured cortical cells cultured with microglia (PDF 43 KB) [file 11033_2020_6092_MOESM2_ESM.pdf]

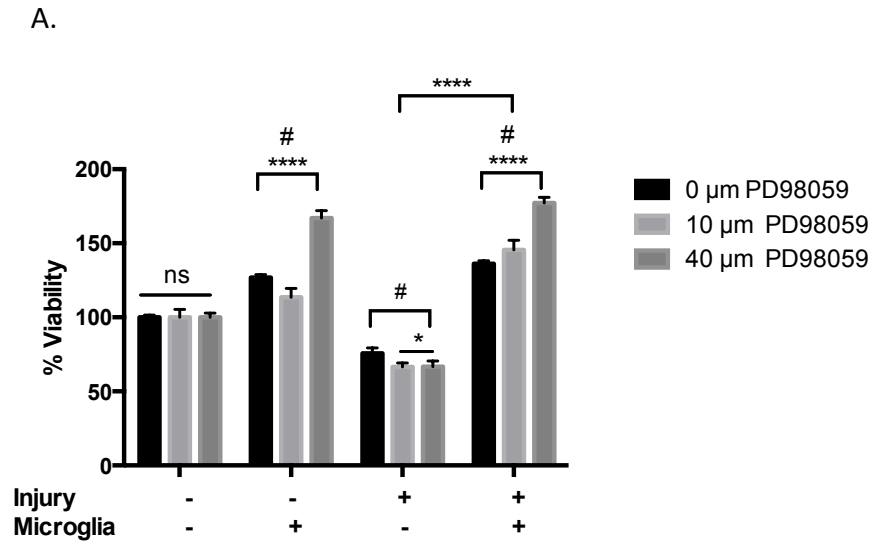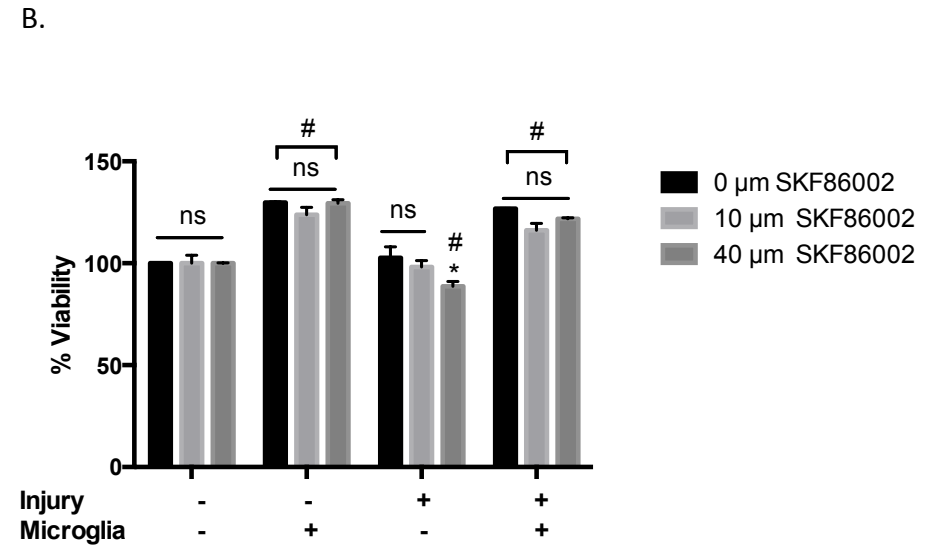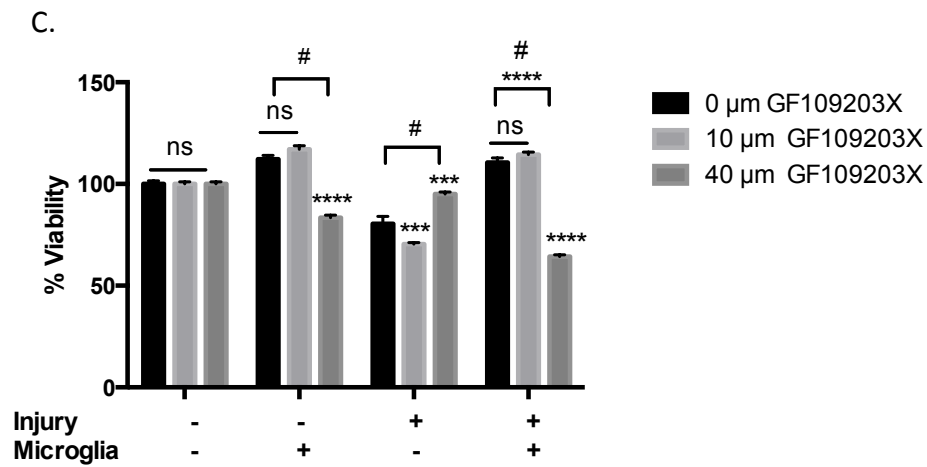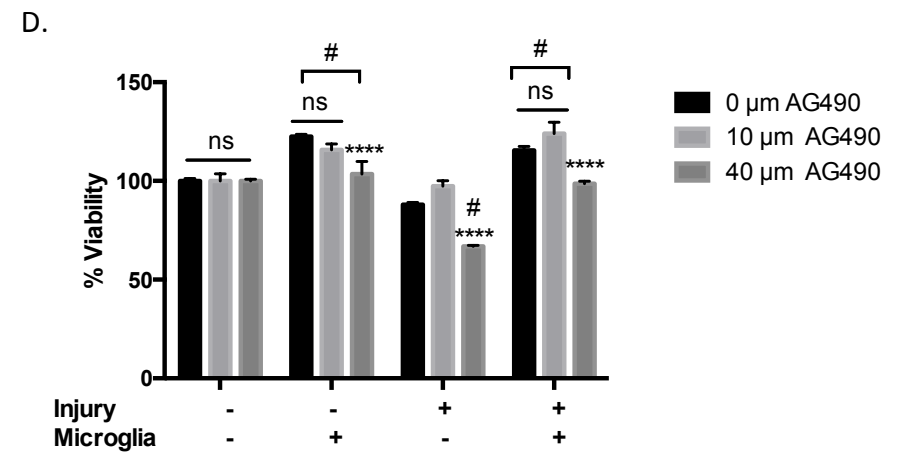

Supplement: Supplementary file 3 — Supplementary file3 Online Resource 3 BV2 microglia co-cultured with cortical cells enhances neurogenic protein expression A) Immunofluorescence of Nestin+ (red), α-internexin+ (green), NeuN+ (red) and GFAP+ (green) cells in injured cortical cells co-cultured without or with microglia. DAPI (blue) was used to observe nuclei of all cultured cells. Scale bar represents 500 µm. All images were acquired with a 20X Leica objective. B) Representative western blot images of protein from injured cortical cultures in control media without microglia (-) or injured cortical cultures with microglia. GAPDH was used as a total protein loading control. C) Quantification of relative fluorescence of neurogenic protein expression. Three separate fields within injured cortical cultures were evaluated for protein expression using immunofluorescent measurement software to determine the fluorescence intensity units for each protein marker. Averaged fluorescent intensity data from injured cortical cell cultures were normalized and set equal to 1 to determine relative fluorescent intensity units (RFU). Fold change in RFU in injured cortical cultures with microglia was determined and multiple Student T Tests were performed to determine significance. Error bars represent SEM. Significance is **p<0.01, ***p<0.001, ****p<0.0001 (PDF 159 KB) [file 11033_2020_6092_MOESM3_ESM.pdf]

AKT (kDa)

L. 1. 2. 3. 4.

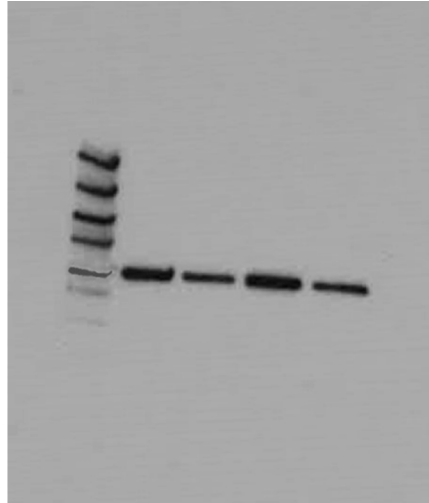

P-AKT ( kDa)

L. 1. 2. 3. 4.

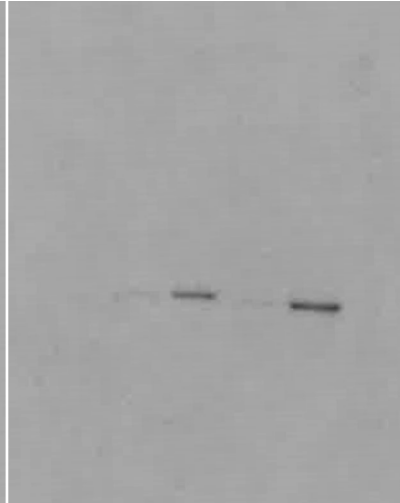

Supplement: Supplementary file 4 — Supplementary file4 Online Resource 4 Multiplex ELISA and RT-PCR analyses of inflammatory cytokine protein and mRNA following co-culture with uninjured or injured cortical cells. IL-1, IL-1, IL-2, IL-4, IL-3, IL-6, IL-10, IL-12, 1L-17 and GM-CSF were either undetectable in all culture conditions or were not significantly different when detected in co-cultures of uninjured and injured cortical cells with microglia. Error bars represented SEM. Two-way ANOVA followed by Tukey’s multiple comparisons test was performed to determine the significance (PDF 274 KB) [file 11033_2020_6092_MOESM4_ESM.pdf]

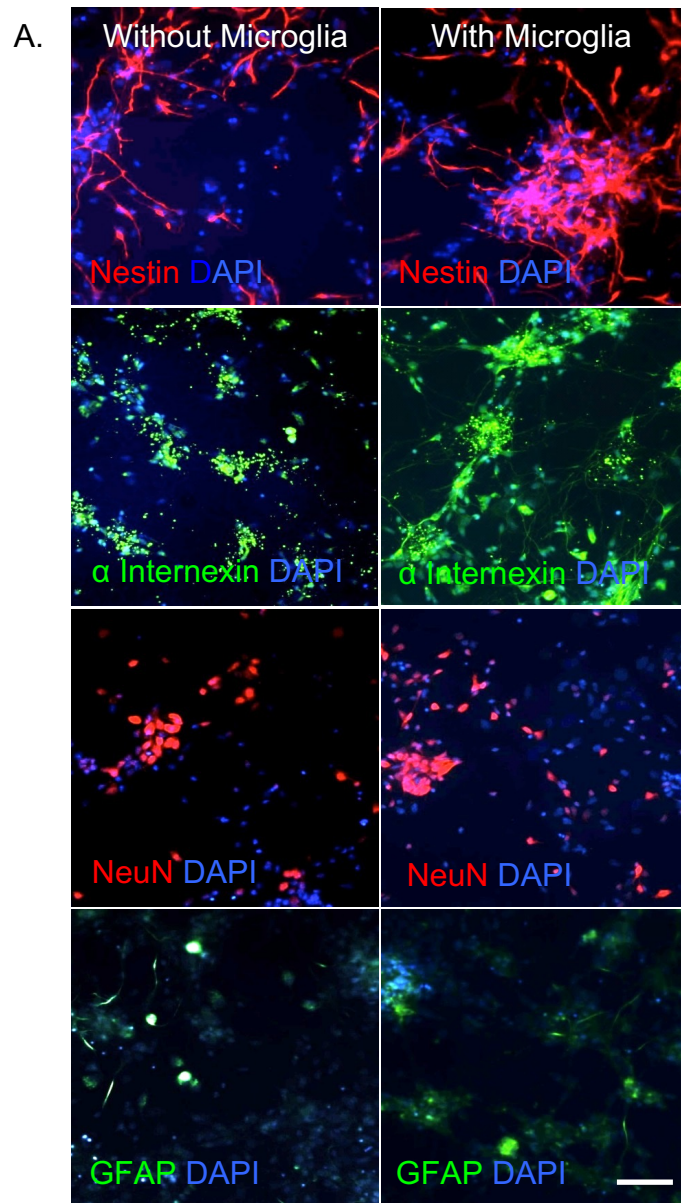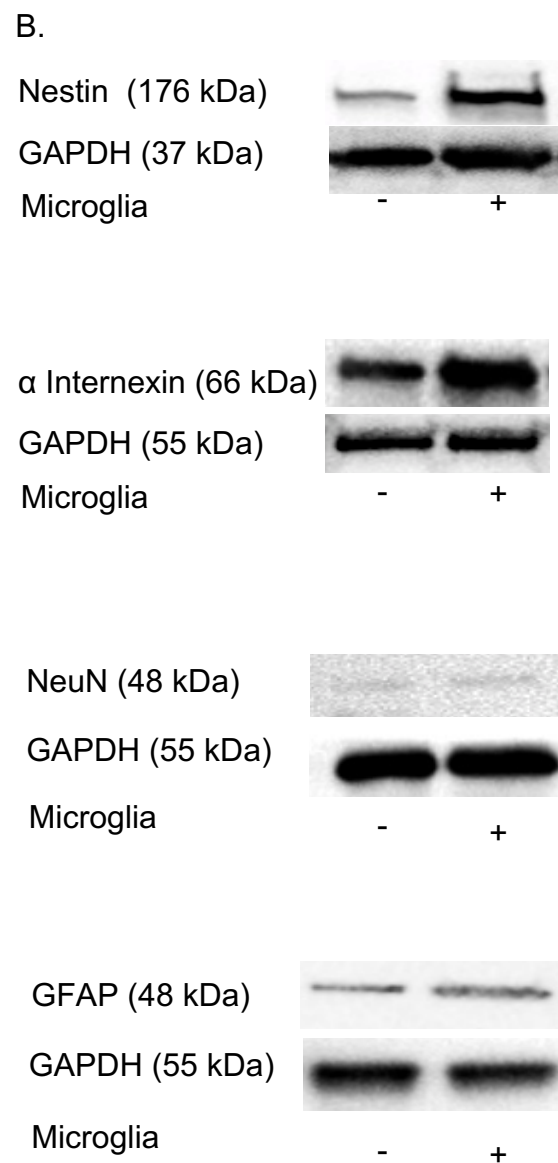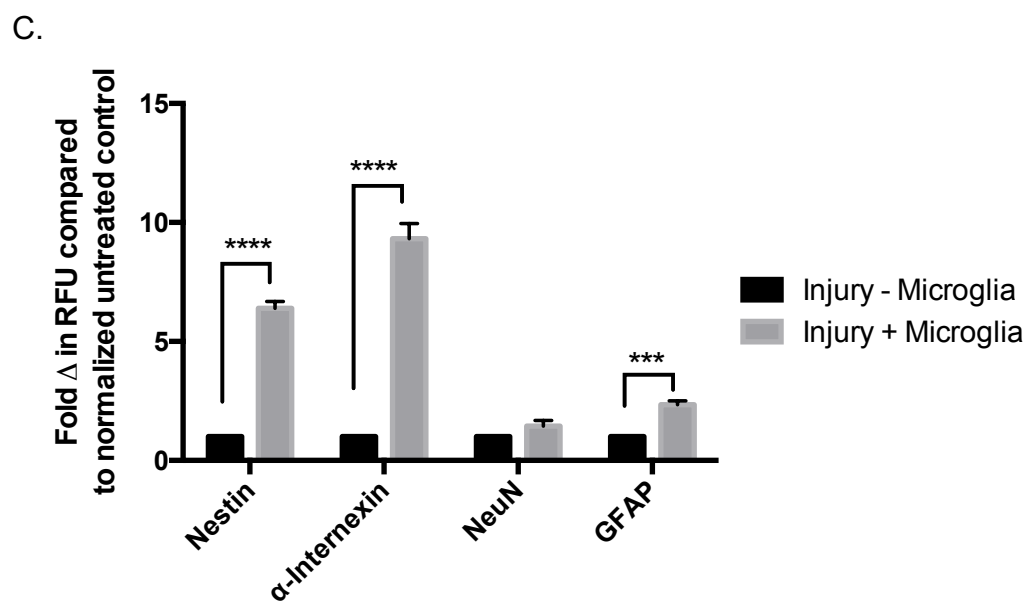

Supplement: Supplementary file 5 — Supplementary file5 Online Resource 5 Inhibitors of MEK, p38, or PKC intracellular signaling pathways do not specifically inhibit EOC2 microglial-enhanced cortical cell viability. A-D) Quantification of MTT viability assays were performed in triplicate experiments using three biological replicates of primary cortical cells. Average OD 595 nm values were converted to percent viability and are shown with error bars representing SEM. Inhibitors were applied at 0, 10, and 40 µM. A) Quantification of viability of uninjured and injured cortical cells alone or in co-culture with microglia in the presence of MEK inhibitor PD98059. B) Quantification of viability of uninjured and injured cortical cells alone or in co-culture with microglia in the presence of p38 MAPK inhibitor SKF86002 C) Quantification of viability of uninjured and injured cortical cells alone or in co-culture with microglia in the presence of PKCα/βI/βII/γ inhibitor GF109203X. D) Quantification of viability of uninjured and injured cortical cells alone or in co-culture with microglia in the presence of Janus Kinase 2 inhibitor AG490. Two-way ANOVA was used to determine significance of the inhibitors and treatment groups. For PD98059 the F value between treatment groups was 16.96. For GF109203 the F value between treatment groups was 81.74. For SKF86002 the F value between treatment groups was 4.83. For AG490 the F value between treatment groups was 11.28. *p,0.05, **p<0.01, ***p<0.001, ****p<0.0001, # indicates that percent viability was significantly different from that of control, uninjured cortical cells alone, ns indicates not significant (PDF 1920 KB) [file 11033_2020_6092_MOESM5_ESM.pdf]

A.

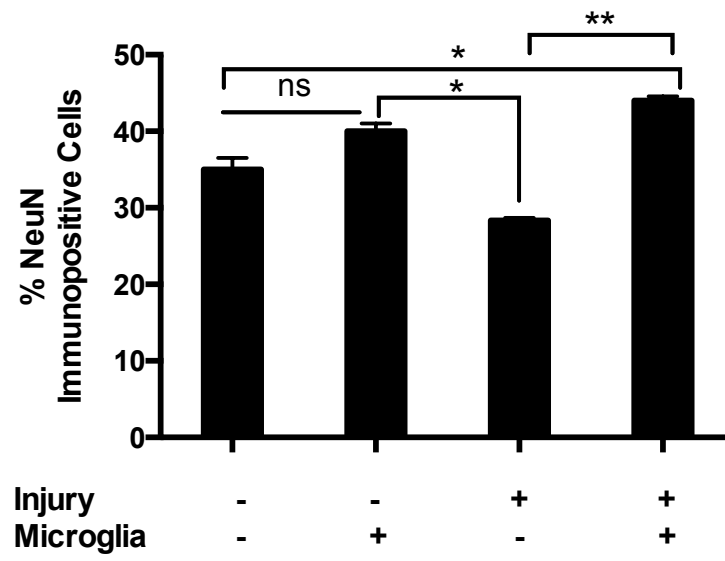

B.

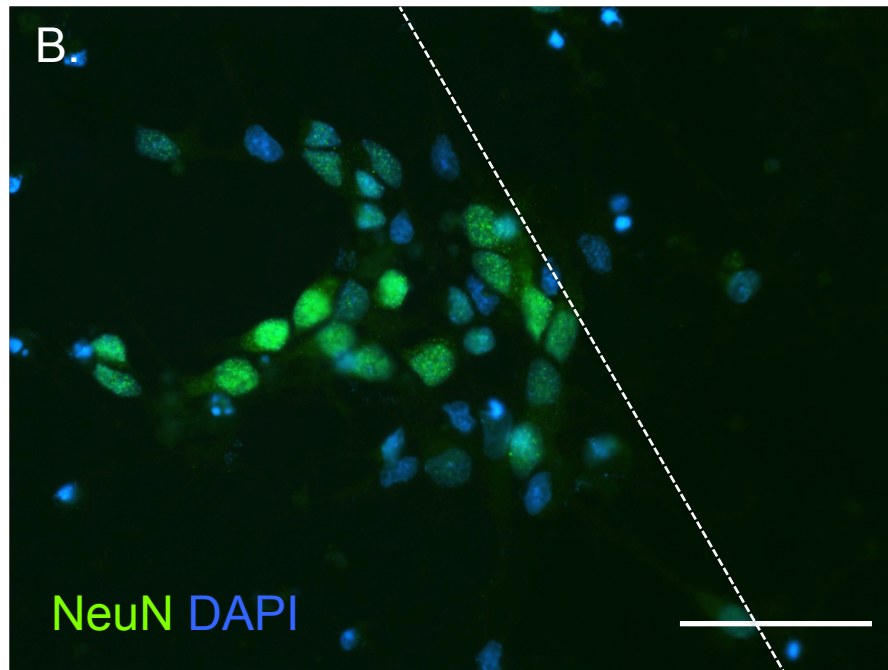

C.

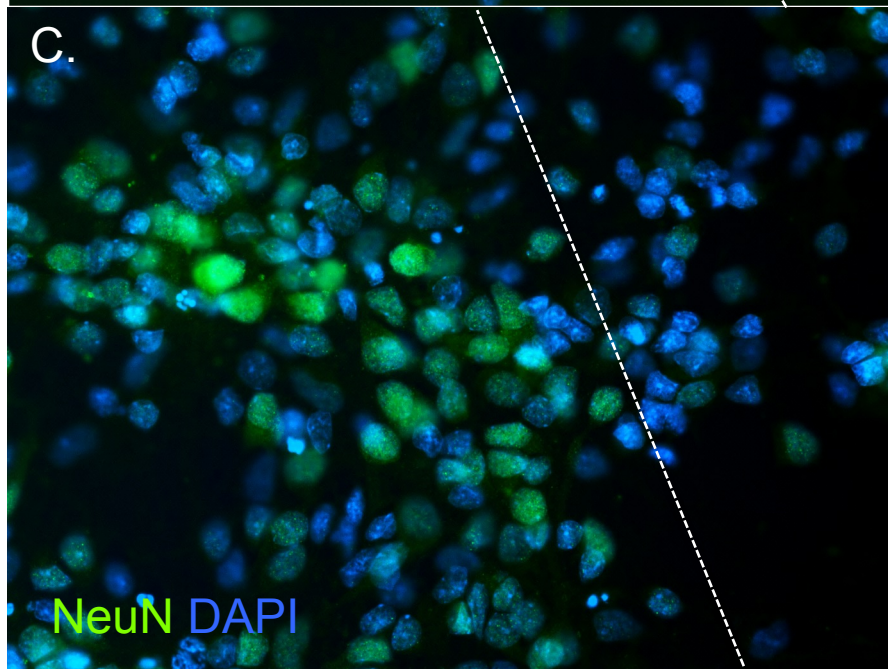

Supplement: Supplementary file 6 — Supplementary file6 Online Resource 6 Western blot images of AKT and phosphorylated AKT (pAKT) shown in Fig 8. Lanes are designated as L: ladder, Lane 1: Protein from uninjured cortical cells cultured without microglia., Lane 2: Protein from uninjured cortical cells cultured with microglia., Lane 3: Protein from injured cortical cells cultured without microglia., Lane 4: Protein from injured cortical cells cultured with microglia (PDF 1802 KB) [file 11033_2020_6092_MOESM6_ESM.pdf]
